# Multiple cause of death during the COVID-19 pandemic: a population study In Colombia and Brazil

**DOI:** 10.3389/ijph.2026.1609429

**Published:** 2026-05-18

**Authors:** Doris Durán, Mabel Carabali, Usama Bilal, Belinda Nicolau, Jay S. Kaufman

**Affiliations:** 1 Department of Epidemiology Biostatistics and Occupational Health, McGill University, Montreal, QC, Canada; 2 Population Health Laboratory (#PopHealthLab), University of Fribourg, Fribourg, Switzerland; 3 Swiss School of Public Health+, Zürich, Switzerland; 4 Department of Epidemiology and Biostatistics, Drexel Dornsife School of Public Health, Philadelphia, PA, United States; 5 Faculty of Dentistry, McGill University, Montreal, QC, Canada

**Keywords:** chronic diseases, COVID-19, death certificates, epidemiology, mortality, Latin America

## Abstract

**Objectives:**

To assess differences in age-standardized disease-specific mortality rates derived from the underlying cause of death (UCOD) versus multiple cause of death (MCOD) data in two Latin American countries during the COVID-19 pandemic.

**Methods:**

Using all death certificates for residents of Brazil (n = 6,207,785) and Colombia (n = 1,180,880) from 2019 through 2022, we extracted UCOD and all contributing causes, assigned weights (50% to UCOD, remainder equally among contributing causes), and calculated annual age- and sex-standardized mortality rates for neoplasms, circulatory diseases, diabetes mellitus (DM), and non-COVID communicable diseases. We then computed rate differences and rate ratios contrasting MCOD with UCOD estimates.

**Results:**

MCOD-DM mortality exceeded UCOD by up to 70%. In Colombia, MCOD–CVD surpassed UCOD during 2020–2021, corresponding to approximately 1,950 extra male and 1,560 extra female CVD deaths in 2021. Discrepancies for neoplasms and other communicable diseases were smaller and stable.

**Conclusion:**

These findings demonstrate that MCOD methods reveal substantial underestimation of diabetes- and cardiovascular-related mortality, underscoring the value of MCOD surveillance for public health planning.

## Introduction

Mortality statistics, derived from legally mandated death certificates, are central to public health planning and population health monitoring. A key component of the death certificate is the medical certification of death, which generally includes two parts. Part I records the sequence of events leading directly to death, beginning with the immediate cause and listing any antecedent/intermediate causes. From this, the underlying cause of death (UCOD), the disease or injury that initiated the chain of events, is selected. Part II lists other significant conditions that contributed to the death but were not part of the causal sequence (see Supplementary Material 1 for an example). The International Classification of Diseases (ICD-10) provides rules for selecting the UCOD and assigns it a specific code for statistical purposes [1].

Mortality statistics use the UCOD as the cause of death to understand cause-specific mortality and design public health policies [1, 2]. However, attribution of the cause of death to a single disease or event, the UCOD, may hinder surveillance efforts. Concurrent diseases (e.g., non-communicable, neurodegenerative) may interact to initiate death, and the decision to select one over others can be arbitrary or subjective [2–6]. Specifically, the burden of non-communicable diseases that often contribute to death but are not selected as the UCOD, for example, diabetes, is underestimated in traditional mortality statistics [7–10].

These limitations in cause-of-death attribution became especially visible during the COVID-19 pandemic. In early 2020, the World Health Organization (WHO) created new rules to define a death due to COVID-19, and one of the most consequential decisions was that, for a death in a subject with a clinically compatible case of COVID-19, the death could not be attributed to another cause [11, 12]. This directive heightened concerns around the singular selection of the UCOD, as it may have obscured the role of other chronic conditions that contributed to death [13, 14]. Indeed, concerns that mortality statistics were affected by the selection of the cause of death and coding directives during the pandemic have led several authors to suggest further analysis using multiple cause of death (MCOD) approaches. This analysis, which accounts for health conditions reported in the death certificate (e.g., diabetes, cancer, or cardiovascular disease), can help elucidate the underestimated contribution of non-UCOD conditions, providing a better understanding of the causes and patterns of death in the population [6].

The extent of impacts on cause-specific mortality has been recently studied in high-income countries [8, 15, 16]. However, assessments in Latin America remain scarce, despite the substantial toll of the COVID-19 pandemic in the region [17]. In Brazil and Colombia, two of the largest and most affected countries in the region, excess mortality rose sharply during 2020–2021. Brazil recorded nearly 800,000 excess deaths, the fourth highest globally [18], and in Colombia, excess mortality exceeded 40% above baseline [18, 19]. Both countries maintain national vital statistics systems that allow detailed analysis of causes of death, and together they represent a substantial share of the region’s population and mortality burden, making them key settings for understanding cause-specific mortality patterns in Latin America.

In this study, we use vital statistics data from Brazil and Colombia to assess cause-specific mortality from 2019 to 2022. We will compare UCOD and MCOD approaches to describe mortality patterns in Neoplasms (CA), Circulatory system diseases (CVD), Communicable diseases (CDs), and Diabetes Mellitus (DM) in men and women.

## Methods

### Target population

All residents of Colombia and Brazil from 2019 to 2022.

### Data sources

For Colombia, individual death records data were accessed from the open data portal of the National Administrative Department of Statistics (DANE in Spanish). For Brazil, we accessed the Mortality System Database through the Data Science Platform applied to Health (PCDaS in Portuguese) [20]. PCDaS extracts the death data from the DATASUS (Ministry of Health) and conducts a first data cleaning protocol, such as replacing invalid values and separating information into new variables (details can be seen in the SIM documentation on the PCDaS website) [21]. For both countries, death records included the underlying cause of death (UCOD) and contributing causes, as documented in Part II of the death certificate. We used national population projections by age and sex derived from the 2018 Census in Colombia, which cover the period 2018–2070, and from the 2022 Census in Brazil, which covers the period 2000–2070. In both countries, projections are produced using the demographic components method, incorporating trends in fertility, migration, and mortality. For Brazil, we also used retroprojections for 2018–2021. Our analysis focuses on the period 2019–2022 [22, 23].

### Outcome

Our outcome was mortality rates from Neoplasms, Circulatory system diseases (CVD), Communicable diseases (CD), and Diabetes mellitus (DM) as defined in the Cause of death list 6/67 from PAHO/WHO (details in Supplementary Material 2). We excluded COVID-19 deaths from the Communicable diseases group. Mortality rates were estimated in two ways: (i) using the traditional UCOD, i.e., considering all deaths with a specific disease as the UCOD; and (ii) using a weighted MCOD approach, where half of the weight was assigned to the UCOD and the other half was divided equally among all other causes reported in Part II of the death certificate (details in Supplementary Material 3). This 50% allocation was chosen to prioritize the underlying condition as the primary driver of mortality while formally accounting for the contribution of comorbid conditions. The assignment of partial weights ensures that each decedent is counted once [4, 24], such that the total contribution of each death is fixed and additional reported causes redistribute rather than increase the total weight assigned across causes. We estimated MCOD by considering all weighted cause-specific deaths. Both outcomes were estimated by age-sex strata each year.

### Sensitivity analysis

We applied a different weighting scheme where the total weight was divided equally between the UCOD and the contributing causes. This strategy evaluates a conceptualization where all reported causes are considered equally important for the death to occur, allowing us to assess the extent to which prioritizing the UCOD might under-represent certain conditions during periods of high cause competition.

### Missing data

Death records with missing information for sex corresponded to 0.02% in Colombia and 0.04% in Brazil. Likewise, for age, 0.01% was missing in Colombia and 0.24% in Brazil (details in Supplementary Material 4). Single imputation with a logistic regression was used for sex in both countries. Similarly, for age, we used a multinomial logistic regression and predictive mean matching for Colombia and Brazil, respectively. The imputation models were conditional on all other complete or imputed variables in the dataset.

### Statistical analyses

We estimated age- and sex-specific mortality rates by cause of death. Mortality rates were then standardized (ASMR) using the WHO 2000–2025 standard population and stratified by sex (age-specific results available in Supplementary Materials 5–12). We conducted analyses separately using the UCOD and a weighted MCOD approach, and to quantify differences between approaches, we calculated annual standardized rate differences and standardized rate ratios of the contrast between MCOD versus UCOD. These indicators can be interpreted as measures of discrepancy between the two approaches. Thus, values closer to the null, 0 for the absolute difference and 1 for the relative difference, indicate that both methods yield similar results, i.e., the MCOD approach does not add information to the UCOD-derived knowledge. We additionally observed how these indicators changed over time from 2019 through 2022, considering the onset of the COVID-19 pandemic in March 2020.

As recommended in the literature, we excluded deaths with ICD-10 codes from the Chapter XVIII: Symptoms, signs and abnormal clinical and laboratory findings, not elsewhere classified (ICD-10 codes R00-R99). They do not provide information for the UCOD and cannot be incorporated into the weighting strategies [4]. We also performed a secondary monthly analysis, which is available in Supplementary Materials 13, 14. All analyses were done in STATA V.18, and the code is available in the Supplementary Material.

## Results

### Overall characteristics

Table 1 shows the main characteristics of decedents in Brazil (n = 6,207,785) and Colombia (n = 1,180,880) during 2019–2022. In both countries, 2021 had the highest number of deaths. Most decedents were men (e.g., for 2020 in Colombia, 43.0% were women and 57.0% men; and for Brazil, 43.8% were women and 56.2% were men).

**TABLE 1:** Characteristics of decedents by year. Colombia and Brazil, 2019-22. (Multiple cause of death during the COVID-19 pandemic: a population study In Brazil and Colombia).

	Colombia				Brazil			
	2019	2020	2021	2022	2019	2020	2021	2022
N (%)	240,504	297,346	359,441	283,589	1,329,447	1,538,054	1,814,047	1,526,237
Sex								
Men	132,387 (55.0)	169,511 (57.0)	203,384 (56.6)	157,123 (55.4)	734,447 (55.2)	863,952 (56.2)	1,005,245 (55.4)	835,188 (54.7)
Women	108,117 (45.0)	127,835 (43.0)	156,057 (43.4)	126,466 (44.6)	595,000 (44.8)	674,102 (43.8)	808,802 (44.6)	691,049 (45.3)
Age groups (years)								
<20	10,771 (4.5)	9,427 (3.2)	10,268 (2.9)	10,470 (3.7)	44,990 (3.4)	40,503 (2.6)	40,584 (2.2)	42,412 (2.8)
20-29	11,827 (4.9)	11,932 (4.0)	14,197 (3.9)	13,324 (4.7)	48,924 (3.7)	53,010 (3.4)	56,868 (3.1)	51,792 (3.4)
30-39	11,312 (4.7)	12,556 (4.2)	16,238 (4.5)	13,014 (4.6)	60,011 (4.5)	68,180 (4.4)	85,218 (4.7)	63,715 (4.2)
40-49	12,891 (5.4)	15,927 (5.4)	22,488 (6.3)	14,739 (5.2)	89,078 (6.7)	106,592 (6.9)	144,085 (7.9)	101,113 (6.6)
50-59	22,174 (9.2)	29,559 (9.9)	39,723 (11.1)	24,011 (8.5)	155,663 (11.7)	184,381 (12.0)	240,989 (13.3)	167,841 (11.0)
60-69	36,350 (15.1)	50,103 (16.9)	64,193 (17.9)	42,039 (14.8)	237,323 (17.9)	286,592 (18.6)	350,487 (19.3)	269,601 (17.7)
70-79	49,040 (20.4)	64,438 (21.7)	76,563 (21.3)	58,285 (20.6)	282,642 (21.3)	335,446 (21.8)	390,740 (21.5)	333,825 (21.9)
80+	86,139 (35.8)	103,404 (34.8)	115,771 (32.2)	107,707 (38.0)	410,816 (30.9)	463,350 (30.1)	505,076 (27.8)	495,938 (32.5)
Causes of death (CoD)								
Neoplasms	48,880 (20.3)	49,107 (16.5)	49,225 (13.7)	49,949 (17.6)	235,301 (17.7)	229,300 (14.9)	235,805 (13.0)	244,009 (16.0)
Circulatory system diseases	75,826 (31.5)	84,673 (28.5)	94,432 (26.3)	92,084 (32.5)	364,132 (27.4)	357,741 (23.3)	382,507 (21.1)	400,154 (26.2)
Diabetes Mellitus	7,967 (3.3)	10,198 (3.4)	10,268 (2.9)	8,703 (3.1)	66,710 (5.0)	75,712 (4.9)	78,258 (4.3)	75,838 (5.0)
Communicable diseases	16,850 (7.0)	16,223 (5.5)	18,705 (5.2)	16,619 (5.9)	143,030 (10.8)	124,772 (8.1)	132,583 (7.3)	158,755 (10.4)
Ill-defined	2,739 (1.1)	3,495 (1.2)	3,634 (1.0)	3,704 (1.3)	74,972 (5.6)	90,345 (5.9)	94,134 (5.2)	82,597 (5.4)
COVID-19	1 (0.0)	51,271 (17.2)	90,801 (25.3)	13,421 (4.7)	4 (0.0)	212,706 (13.8)	424,461 (23.4)	65,764 (4.3)
Other	88,241 (36.7)	82,379 (27.7)	92,376 (25.7)	99,109 (34.9)	445,298 (33.5)	447,478 (29.1)	466,299 (25.7)	499,120 (32.7)
Part 2 completion: No. of CoDs in Part 2								
0	160,309 (66.7)	174,949 (58.8)	197,483 (54.9)	151,895 (53.6)	846,850 (63.7)	891,410 (58.0)	1,039,937 (57.3)	874,552 (57.3)
1	58,221 (24.2)	79,427 (26.7)	97,665 (27.2)	73,810 (26.0)	260,988 (19.6)	320,986 (20.9)	382,836 (21.1)	324,322 (21.2)
2	16,287 (6.8)	24,766 (8.3)	36,443 (10.1)	36,936 (13.0)	193,932 (14.6)	277,720 (18.1)	333,816 (18.4)	276,937 (18.1)
3+	5,687 (2.4)	18,204 (6.1)	27,850 (7.7)	20,948 (7.4)	27,677 (2.1)	47,938 (3.1)	57,458 (3.2)	50,426 (3.3)

The number of causes of death listed in Part II of the death certificate increased over time. The proportion of death certificates with no causes in Part II decreased from 66.7% to 53.6% in Colombia and from 63.7% to 57.3% in Brazil between 2019 and 2022, leading to more certificates with two or more causes in Part II. These patterns were consistent across sex and age groups. Although older age groups had a lower proportion of certificates with no information in Part II, reflecting higher comorbidity, the temporal increase in reporting in Part II was observed across all strata (Supplementary Material 17).

The emergence of COVID-19 as a cause of death in 2020 produced a decrease in the annual proportion of deaths from other causes, a trend that further intensified in 2021. Most disease groups returned to pre-pandemic proportions in 2022.

### Cause-specific mortality trends - UCOD approach

**FIGURE 1:**
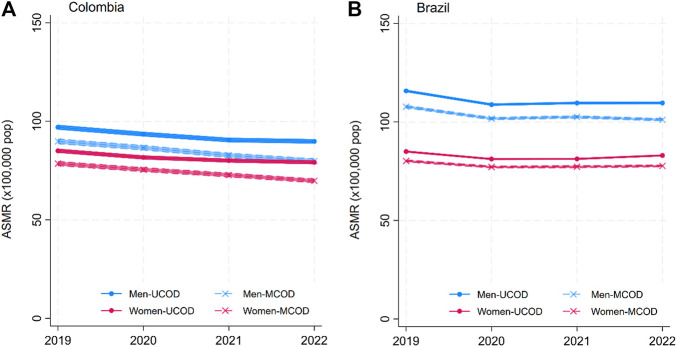
Annual age-standardized mortality rates (ASMR) for neoplasms by sex: underlying cause of death (UCOD) and weighted multiple cause of death (MCOD). (**(A)** Brazil and **(B)** Colombia, 2019–22). (Width of the lines corresponds to 95% confidence intervals). (Multiple cause of death during the COVID-19 pandemic: a population study In Brazil and Colombia).

**FIGURE 2:**
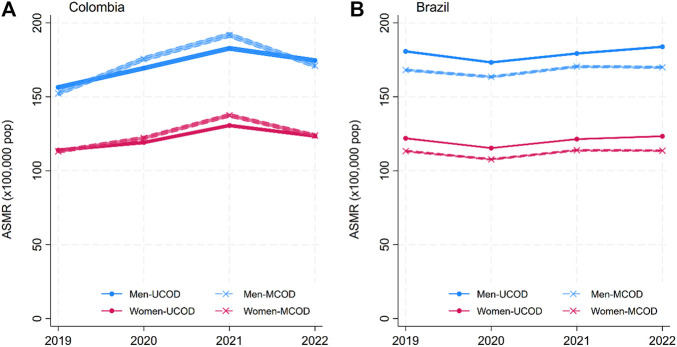
Annual age-standardized mortality rates (ASMR) for circulatory system diseases (CVD) by sex: underlying cause of death (UCOD) and weighted multiple cause of death (MCOD). (**(A)** Brazil and **(B)** Colombia, 2019–22). (Width of the lines corresponds to 95% confidence intervals) (Multiple cause of death during the COVID-19 pandemic: a population study In Brazil and Colombia).

**FIGURE 3:**
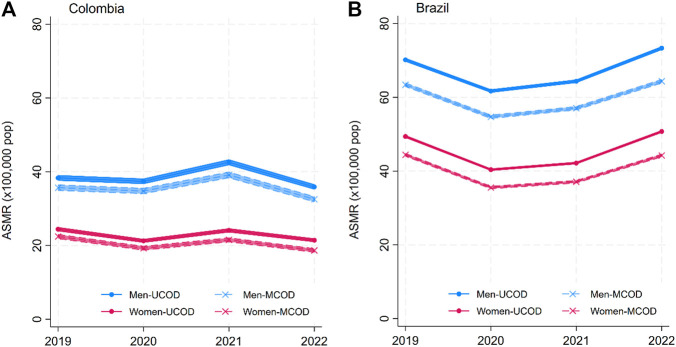
Annual age-standardized mortality rates (ASMR) for Communicable Diseases (CDs) by sex: underlying cause of death (UCOD) and weighted multiple cause of death (MCOD). (**(A)** Brazil and **(B)** Colombia, 2019–22). (Width of the lines corresponds to 95% confidence intervals). (Multiple cause of death during the COVID-19 pandemic: a population study In Brazil and Colombia).

**FIGURE 4:**
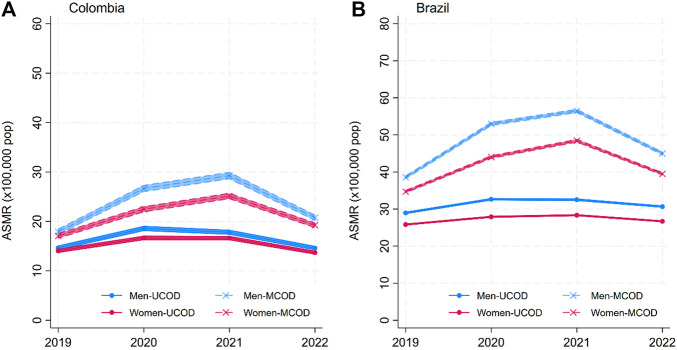
Annual age-standardized mortality rates (ASMR) for Diabetes Mellitus (DM) by sex: underlying cause of death (UCOD) and weighted multiple cause of death (MCOD). ((A) Brazil and (B) Colombia, 2019–22). (Width of the lines corresponds to 95% confidence intervals). (Multiple cause of death during the COVID-19 pandemic: a population study In Brazil and Colombia).

The solid lines in Figures 1–4 show the age-standardized mortality rates (ASMR) for neoplasms, CVD, DM, and CDs for men and women, based on the traditional UCOD approach. While there were some changes (generally increases) in mortality rates in 2020–2021, by 2022, all disease-specific rates had returned to levels similar to 2019. Mortality was slightly higher for men and higher in Brazil than in Colombia.

### MCOD versus UCOD

The dashed lines in Figures 1–4 display the MCOD mortality patterns. Mortality rates were consistently lower for Neoplasms and Communicable diseases in both countries and for CVD in Brazil. However, DM in both countries and CVD in Colombia had higher MCOD-mortality rates. This discrepancy is further accentuated in 2021.

**FIGURE 5:**
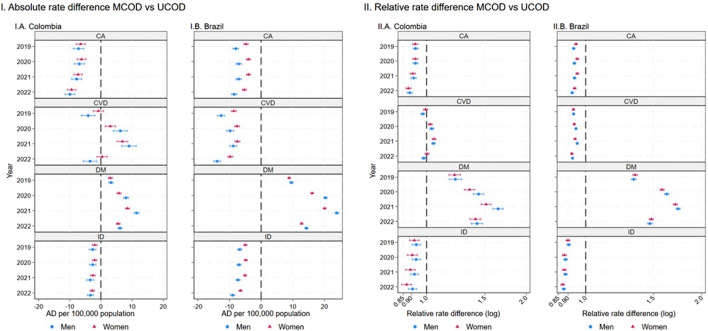
Absolute and relative differences for weighted multiple cause of death (MCOD) versus underlying cause of death (UCOD) mortality rates for Neoplasms, Circulatory System Diseases, Communicable diseases, and Diabetes Mellitus by sex. Brazil and Colombia, 2019-22. Horizontal lines correspond to 95% pointwise confidence intervals. Values above (below) the null of 0 in **(I)** and of 1 in **(II)** represent mortality rates that were higher (lower) for the MCOD method as compared to the UCOD method. Abbreviations: CA, Neoplasms, CVD, Circulatory System Diseases, CD, Communicable Diseases, and DM, Diabetes Mellitus. AD, absolute difference; RD, relative difference. (Multiple cause of death during the COVID-19 pandemic: a population study In Brazil and Colombia).

Figure 5 shows the absolute and relative differences between the MCOD and UCOD mortality rates. The MCOD method shows fewer deaths per 100,000 people for neoplasms and CD in both countries but varies greatly for DM and CVD. This is also visible in terms of relative differences. Supplementary Tables S3, S4 in the Supplementary Material show disease-specific indicators for Colombia and Brazil, respectively.

The MCOD method consistently showed higher DM mortality, as compared to UCOD even in 2019, with 3.25 (95% confidence interval (95%CI) 2.54; 3.96) more deaths per 100,000 men and 3.02 (95%CI 2.39; 3.65) more deaths per 100,000 women in Colombia. Likewise, in Brazil in 2019, the MCOD method reports 9.61 (95%CI 9.11; 10.10) more deaths per 100,000 men and 8.87 (95%CI 8.46; 9.28) per 100,000 women as compared to UCOD. This discrepancy is further magnified during the COVID-19 pandemic. In Colombia, the 2021 rate difference is 11.47 (95%CI 10.64; 12.29) and 8.52 (95%CI 7.82; 9.22) for men and women, respectively. This corresponds to 64% (95%CI 59; 70) higher diabetes related mortality for men, and 51% (95%CI 46; 57) for women when using the MCOD method during the worst year of the pandemic (Figure 5, Panel IIA). Similarly, in Brazil in 2021, DM-related mortality was 74% (95%CI 71; 76) and 71% (95%CI 69; 73) higher for women when using the MCOD method.

For CVD, MCOD mortality in Colombia was lower than UCOD in 2019 and 2022, with 4.07 (95%CI −6.25; −1.90) and 3.45 (95%CI −5.65; −1.25) fewer deaths per 100,000 men, for each year respectively. In 2021, however MCOD showed 9.05 (95%CI 6.73; 11.36) more deaths per 100,000 men. This shift was also visible for women, meaning that the contribution of CVD to mortality was higher than what was captured with the traditional UCOD mortality method in both men and women. In Brazil, however, MCOD consistently showed lower CVD mortality rates as compared to UCOD during the entire study period and for both sexes.

The mortality reported with the MCOD method for CDs and neoplasms was consistently lower than the mortality reported with the UCOD method for both countries. For neoplasms mortality, the absolute gap between the methods in Colombia changed from −7.17 (95%CI −8.88; −5.46) fewer deaths for men in 2019, to −9.93 (95%CI −10.73; −8.05) in 2022. While in both countries the absolute and relative differences for MCOD/UCOD methods for CDs mortality are very stable in the period of study, the age-specific results (Supplementary Materials 5–12) show the largest heterogeneity between age-groups. CDs mortality rates decreased in the youngest (<20 years old) and oldest groups (>=80 years) in 2020 and 2021 for both countries.

Our secondary monthly analysis (available in Supplementary Materials 13,14) shows increases in mortality from CVD, CDs and DM during 2020 and 2021. Specifically in Colombia, while this is visible with UCOD and MCOD, the latter showed, similar to in the main analysis, higher mortality for CVD and DM between June of 2020 and June of 2021. For Brazil, however, only DM-MCOD mortality was higher than UCOD the entire period of study, but also it doubled the number of deaths per 100,000 reported with the traditional method during June of 2020 and June of 2021, which also corresponds to high COVID-19 mortality.

In the sensitivity analysis to test the weighting attribution, we used a different strategy that divides the total weight representing one death, in equal parts among every disease or condition reported as a contributing cause. The results available in Supplementary Material 16 are comparable to the main analysis as it also considers distribution of partial weights.

## Discussion

Our work analyzed cause-specific mortality patterns for four groups of diseases in Brazil and Colombia from 2019 to 2022, comparing underlying‐cause (UCOD) and multiple‐cause of death (MCOD). We observed changes in mortality trends, particularly in 2020 and 2021, influenced by the COVID-19 pandemic. Across all causes examined, mortality estimates from UCOD and MCOD approaches differed in magnitude, with variation depending on the causes of death and by age group. In general, we found that diabetes mortality was higher using the MCOD method, cancer and CDs were higher using the UCOD method, and CVD varied by country and time.

For diabetes, we identified that MCOD-attributed mortality exceeded UCOD estimates in every year of the study in both countries. Similar underestimation has been reported in pre-pandemic studies in North America, where the UCOD approach also failed to capture the full burden of DM-related mortality. In the US, between 2003 and 2016, only 3% of deaths in people over 25 years had diabetes as UCOD, but this was 6.7% as MCOD [9]. Likewise, when UCOD from mortality statistics was compared to national surveys, the 3.6% of mortality attributed to Diabetes increased to 5.1% by considering diabetes as a contributing cause [10]. Furthermore, studies in Australia, with calculations of the burden of disease through years of life lost (YLL) with weighted MCOD methods, yielded 31% higher YLL for DM, compared to the YLL with the UCOD [25].

Additionally, for DM mortality, there was a large widening of the gap between MCOD and UCOD of up to 71% during 2020 and 2021. This suggests that diabetes played a substantial role in the burden of mortality during these years, one which is significantly underestimated by the traditional UCOD approach and by the competition introduced with the decisions of selecting one disease/condition over others. Specifically in Brazil, higher DM mortality was observed when counting any mention of DM in the death certificates, even when COVID-19 deaths were excluded [26], pointing to a greater burden of DM related mortality, and potential underreported COVID-19 deaths. Similarly, in Spain, there was a 25% increase in the reporting of DM in death certificates in 2020 compared to the average in 2018 and 2019, a finding only visible through MCOD analysis [15]. In Northern Italy, DM mortality for adults over 40 years of age increased 19% with the UCOD method and 27% in 2020, with the MCOD analysis. The authors also point out that the largest increase occurred during the last months of 2020, corresponding to the hardest COVID-19 wave in the region [8]. Our findings align with the idea that DM-related deaths increased during peak COVID-19 waves with both UCOD and MCOD estimates.

Overall, MCOD circulatory system diseases (CVD) mortality was consistently lower in Brazil and in 2019 and 2022 in Colombia, reporting fewer CVD deaths (RD < 0 and RR < 1) compared to the UCOD. However, in 2020 and 2021, MCOD surpassed UCOD in Colombia. The CVD-MCOD mortality for men in Colombia during 2021 estimated roughly 1950 more CVD deaths in men and 1,560 more CVD deaths in women that year than the traditional method.

In Brazil, we found stable CVD mortality, with a slight decrease in 2020, as previously reported [27–29]. But a pandemic effect modification is observed on the absolute scale of the MCOD-UCOD comparison, specifically in men, where the absolute difference between methods shrinks in 2020 and 2021, and the relative difference stays constant. This pattern can be explained in different ways. First, CVD may have been reported more often as the UCOD, without any change in how it was reported as MCOD. However, our findings and those of Brant et al. do not support this explanation. In Brazil, Brant et al. observed a 10% increase in CVD mortality in their MCOD analysis [27]. A similar pattern was also reported in the United States [16].

Second, it is possible that CVD was reported less often as a contributing cause, while UCOD stayed the same. Yet this is also unlikely, as CVD-UCOD excess mortality was lower than expected according to Pizzato et al [28]. The third explanation, and the one most supported by our findings and the literature, is that CVD was reported more as a contributing cause and less often as the UCOD in 2020, i.e., both classifications changed. This interpretation is supported by evidence showing that out-of-hospital mortality increased compared to previous years, which may result in less detailed death certificates [27, 30]. The idea of cause competition between COVID-19 and CVD further supports this change. Individuals with chronic conditions have reportedly experienced heightened vulnerability to SARS-CoV-2 and are also more vulnerable to the consequences of a collapsed health system. Thus, while COVID-19 may be selected as the UCOD, the underlying frailty often stems from coexisting diseases, better captured through MCOD methods. Additionally, as referred above, one of the main WHO guidelines in place during the early months of the pandemic required the selection of COVID-19 as the underlying cause if it was present in the certificate, favoring the idea of competition between causes. This is consistent with findings by Bastos et al [31], who reported that, in 2020, COVID-19 was the most common UCOD for deaths in which ischemic heart disease was mentioned.

Neoplasms are usually selected as the underlying cause of death when present on the death certificate, as instructed by ICD-10 rules [4, 24, 32]. Therefore, during the COVID-19 pandemic and following WHO guidelines, a sharp increase in cancer deaths caused by COVID-19 would be another strong example of divergence between MCOD and UCOD methods. Although early concerns were raised about the potential increases in cancer mortality during the pandemic, several studies have reported either no change or lower cancer mortality [28, 29, 33]. We observed similar patterns, with little change in cancer mortality across both countries and for men and women. However, considering a reported reduction of 46% of cervical cancer screening in Colombia in 2020 [34], the effects of the pandemic on cancer mortality may manifest with a lag visible in later years.

Our findings for CDs mortality in Brazil show a decrease during 2020 and 2021, as reported in other countries due to lower respiratory infection deaths (e.g., influenza) during the pandemic years, compared to previous years [15]. However, CDs mortality increased in 2021 in Colombia. While this could be attributed to misclassification of COVID-19 deaths, there were other epidemics in place in Colombia. Our findings by age group show that in Colombia, CDs mortality increased for those between 30 and 49 years of age. Overlapping dengue outbreaks, increasing HIV mortality during the pandemic, and the introduction of Oropuche virus disease can partially explain the findings [35, 36]. Additionally, for both countries, we observed a consistently lower MCOD estimation during the entire period, signaling that communicable diseases are usually selected as the underlying cause of death.

Our results are important for public health surveillance, as they show that the current one-cause-of-death approach is not enough to capture the full scope of cause-specific mortality. Other researchers and we provide evidence of how the UCOD underestimates the full burden of other diseases through an oversimplified conception of disease causality [2, 16, 25, 32, 37–39]. While the current paradigm of mono-causality has contributed to improved population health by identifying conditions for which to prioritize prevention and action efforts, here we present the results of the different ways in which we can measure the burden of each cause of death. This may have consequences in priority setting based on surveillance statistics.

The main limitation of our study is related to data quality and practices around reporting causes of death. While in Latin America, mortality data are collected according to international guidelines, they may not be fully comparable to studies conducted in other settings. MCOD data have only recently become available in Colombia and Brazil and are therefore subject to improvements.

In our study, the proportion of certificates without information in Part II was 66.7% in Colombia and 63.7% in Brazil in 2019, decreasing to 53.6% and 57.3%, respectively, in 2022. This pattern was consistent across sexes and age groups. An increase in the number of contributing conditions may reflect greater multimorbidity, changes in population age structure, or shifts in certification and coding practices. Similar increases in reporting of causes of death have been documented in pre-pandemic settings. For example, a study of death certificates in England and Wales reported increasing numbers of contributing conditions between 2001 and 2017, particularly among older individuals, those in poorer health, and socioeconomically disadvantaged groups [40].

This is relevant because the MCOD approaches rely on the information collected and are particularly sensitive to changes in reporting and coding practices. In our weighted MCOD approach, the total contribution of each death is fixed, such that an increase in the number of reported causes redistributes weight across conditions rather than mechanically increasing cause-specific counts.

At the same time, the number of reported causes reflects not only documentation practices but also the underlying burden of multimorbidity, which may itself have evolved during the study period, including during the COVID-19 pandemic. These mechanisms cannot be disentangled in routine mortality data. Therefore, part of the observed changes in MCOD-based measures, particularly during 2020–2021, may reflect evolving reporting practices in addition to changes in mortality patterns. Notably, the differences between MCOD and UCOD observed prior to the pandemic, such as lower MCOD mortality from neoplasms and communicable diseases and higher MCOD mortality from diabetes mellitus, were sustained in subsequent years. This consistency supports the robustness of our findings and suggests that the main patterns are unlikely to be solely driven by changes in reporting practices.

Additionally, the pandemic may have affected data quality differentially across regions in both countries, and further analyses should address this limitation. Specifically, variations in the national implementation of WHO coding directives or mortality surveillance protocols during the pandemic peak may have varied by country. While our study cannot account for differences in how local registrars prioritized competing causes on death certificates, further qualitative and documentary analyses of these administrative behaviors would be of great value.

The interpretation of our results is also tied to the broader context of data completeness. Death registration completeness is considered high, 96.3% in Brazil and 97.4% in Colombia in 2017 [41], although this varies geographically [42, 43] (as do causes of death). While we did not redistribute ill-defined causes, their impact is mitigated by ongoing quality improvements. Although Brazil has a history of high proportions of ill-defined mortality, several fruitful efforts have been in place to strengthen data quality, and reductions of up to 40% in ill-defined mortality have been achieved nationally [44, 45]. Additionally, both Colombia and Brazil have a high Vital Statistics Performance Index for Quality [VSPI(Q)], classifying them as well-functioning vital statistics systems in 2017 [41].

Despite the limitations discussed above, our study has many strengths. We used methods that have been applied successfully before [4, 24, 25, 32]. Our weighted MCOD maintains each person as the unit of analysis while considering the contributing causes. We chose to allocate half of the weight to the UCOD to acknowledge a condition as more relevant, aligning partially with the UCOD concept, while allowing us to see the contribution of other causes. In our sensitivity analysis, the equal weights strategy challenges the UCOD approach completely, by considering all causes equally important for the death to occur, and also showed that the UCOD method can underestimate mortality.

Additionally, we estimated age-standardized rates for both methods, which set deaths in relation to their own population and control for differences in age structure. This was, moreover, done with population projections based on recent censuses of Colombia and Brazil, ensuring little variation from the census population in the years analyzed.

These methods are proposed as a complement to the traditional approach, and they are becoming more relevant as life expectancy increases, and chronic diseases are the main causes of death. Our study is, to our knowledge, the first to include weighted MCOD methods to estimate cause-specific mortality during the pandemic years in Latin America. Additionally, we used data for all reported deaths in two Latin American countries heavily impacted by the COVID-19 pandemic, which contributes to the knowledge outside of high-income countries.

Our study aligns with recent work to build upon new ways to conceptualize cause(s) of death, when, in general, co-occurring diseases are the leading causes of disease and disability. Most people do not die of an isolated condition in the modern era. Rather, death is often the result of a constellation of metabolic or systemic disorders, many of them preventable, and public health decisions should quantify the whole scope to improve the population’s health. The consideration of multiple causes of death (MCOD) is more informative than only using the UCOD and creates opportunities for improvement. The clear discrepancy between MCOD and UCOD for DM-related mortality in our study is the best example of how the current mortality statistics fall short in informing the population’s burden of diseases.
