# Supplementary material for: Multiple cause of death during the COVID-19 pandemic: a population study In Colombia and Brazil
Source: Int J Public Health. 2026 May 18;71:1609429. doi: 10.3389/ijph.2026.1609429 (PMC13222857; doi:10.3389/ijph.2026.1609429)
Supplement: Supplementary file 1 [file Supplementaryfile1.docx]

**Supplement:** **MULTIPLE CAUSE OF DEATH DURING THE COVID-19 PANDEMIC: A POPULATION STUDY IN COLOMBIA AND BRAZIL**

Table of Contents

[1. Medical certification of death – WHO format 2](#_Toc228866746)

[2. Adapted PAHO 6/67 Mortality Tabulation List 3](#_Toc228866747)

[3. Weighted multiple cause of death 5](#_Toc228866748)

[4. Missing data 6](#_Toc228866749)

[5. Age-specific mortality rates for Neoplasms. Colombia. 7](#_Toc228866750)

[6. Age-specific mortality rates for Circulatory System Diseases. Colombia. 8](#_Toc228866751)

[7. Age-specific mortality rates for Communicable diseases. Colombia. 9](#_Toc228866752)

[8. Age-specific mortality rates for Diabetes Colombia. 10](#_Toc228866753)

[9. Age-specific mortality rates for Neoplasms, Brazil. 11](#_Toc228866754)

[10. Age-specific mortality rates for Circulatory System Diseases. Brazil. 12](#_Toc228866755)

[11. Age-specific mortality rates for Communicable diseases. Brazil. 12](#_Toc228866756)

[12. Age-specific mortality rates for Diabetes. Brazil 14](#_Toc228866757)

[13. Monthly variation in mortality, underlying and weighted multiple cause of death. Colombia. 15](#_Toc228866758)

[14. Monthly variation in mortality, underlying and weighted multiple cause of death. Brazil. 17](#_Toc228866759)

[15. Absolute and relative differences for MCOD versus UCOD mortality 19](#_Toc228866760)

[16. Sensitivity analysis: alternative weighting scheme 21](#_Toc228866761)

[17. Number of causes of death listed in Part II of the death certificate per country, sex and age. 23](#_Toc228866762)

[18. Analysis script 24](#_Toc228866763)

## Medical certification of death – WHO format

| Cause of death | | Approximate interval between onset and death |
| --- | --- | --- |
| I.  **Disease** or condition directly leading to death  **Antecedent causes**  Morbid conditions, if any, giving rise to the above cause, stating the underlying condition last | a)_____________________________________  due to (or consequence of)  b)_____________________________________  due to (or consequence of)  c)_____________________________________  due to (or consequence of)  d)_____________________________________  due to (or consequence of) | _____________  _____________  _____________  _____________ |
| II.  Other significant conditions contributing to the death, but not related to the disease or conditions causing it | _____________________________________  _____________________________________ | _____________  _____________ |

Reproduced from the medical certificate of cause of death recommended by WHO in Medical Certification of Cause of Death. Instructions for Physicians on Use of International Form of Medical Certificate of Cause of Death. Geneva, Switzerland; 1979. Available in: https://iris.who.int/server/api/core/bitstreams/18b123d3-8a32-4c99-8489-cd8db07ea837/content

The MCOD is a section of the death certificate. It is generally structured as shown in the image above, with Part I listing the direct and antecedent causes, from which the underlying cause of death is selected, and Part II recording other contributing conditions. In Brazil and Colombia, deaths are certified by physicians, while the coding of causes of death is performed using Iris software^[[1]](#footnote-1)^, an automated system implemented in 2017 in Brazil and 2019 in Colombia.

## Adapted PAHO 6/67 Mortality Tabulation List

| Category | Subcategory | ICD-10 Codes |
| --- | --- | --- |
| Communicable Diseases | Intestinal infectious diseases | A00–A09 |
|  | Tuberculosis (including sequelae) | A15–A19, B90 |
|  | Certain vector-borne diseases and rabies | A20, A44, A68, A75–A79, A82–A84, A85.2, A90–A91, A95, B50–B57 |
|  | Vaccine-preventable diseases | A33–A37, A80, B05, B06, B16, B17.0, B18.0, B26, B91, P35.0 |
|  | Meningitis | A32.1, A39, A87, B00.3, B01.0, B02.1, B37.5, B38.4, G00–G03 |
|  | Septicemia | A40–A41 |
|  | Infections predominantly sexually transmitted | A50–A64 |
|  | HIV/AIDS | B20–B24 |
|  | Acute respiratory infections | J00–J22 |
|  | Other infectious & parasitic diseases | (remainder of A00–B99) |
| Neoplasms | Malignant neoplasm of stomach | C16 |
|  | Malignant neoplasm of colon | C18 |
|  | Malignant neoplasms of digestive organs & peritoneum (excl. stomach & colon) | C15, C17, C19–C21, C26, C48 |
|  | Malignant neoplasm of liver & intrahepatic bile ducts | C22–C24 |
|  | Malignant neoplasm of pancreas | C25 |
|  | Malignant neoplasms of trachea, bronchus & lung | C33–C34 |
|  | Malignant neoplasms of other respiratory & intrathoracic organs (excl. trachea/bronchus/lung) | C30–C32, C37–C39 |
|  | Malignant neoplasm of breast | C50 |
|  | Malignant neoplasms of uterus | C53–C55 |
|  | Malignant neoplasm of prostate | C61 |
|  | Malignant neoplasms of other genitourinary organs | C51–C52, C56–C57, C60, C62–C68 |
|  | Malignant neoplasms of lymphoid, hematopoietic & related tissue | C81–C96 |
|  | Other malignant neoplasms | (remainder C00–C97) |
|  | Carcinoma in situ, benign & uncertain behavior neoplasms | D00–D48 |
| Diseases of the Circulatory System | Acute rheumatic fever & chronic rheumatic heart diseases | I00–I09 |
|  | Hypertensive diseases | I10–I15 |
|  | Ischemic heart diseases | I20–I25 |
|  | Pulmonary heart disease, diseases of pulmonary circulation & other heart disease | I26–I45, I47–I49, I51 |
|  | Cardiac arrest | I46 |
|  | Heart failure | I50 |
|  | Cerebrovascular diseases | I60–I69 |
|  | Atherosclerosis | I70 |
|  | Other diseases of the circulatory system | I71–I99 |
| Diabetes mellitus | Diabetes mellitus | E10–E14 |
| Others  (including external causes) | Transport accidents: terrestrial | V01–V89, Y85.0 |
|  | Other & unspecified transport accidents | V90–V99, Y85.9 |
|  | Falls | W00–W19 |
|  | Accidents from machinery & sharp objects | W24–W31 |
|  | Accidents from firearms | W32–W34 |
|  | Accidental drowning & submersion | W65–W74 |
|  | Exposure to smoke, fire & flames | X00–X09 |
|  | Accidental poisoning by noxious substances | X40–X49 |
|  | Complications of medical & surgical care (including sequelae) | Y40–Y84, Y88 |
|  | Other accidents (including sequelae) | W20–W23, W35–W64, W75–W99, X10–X39, X50–X59, Y86, Y89.9 |
|  | Intentional self-harm (suicide) (including sequelae) | X60–X84, Y87.0 |
|  | Assault (homicide) (including sequelae) | X85–Y09, Y87.1 |
|  | Legal intervention & war operations (including sequelae) | Y35–Y36, Y89.0–Y89.1 |
|  | Events of undetermined intent (including sequelae) | Y10–Y34, Y87.2 |
|  | Nutritional deficiencies & anemias | E40–E64; D50–D53 |
|  | Mental & behavioral disorders | F00–F99 |
|  | Diseases of the nervous system (excluding meningitis) | G04–G98 |
|  | Chronic lower respiratory diseases | J40–J47 |
|  | Other diseases of upper respiratory tract | J30–J39 |
|  | Pneumoconioses & other lung diseases due to external agents | J60–J70 |
|  | Other respiratory diseases | J80–J98 |
|  | Appendicitis, hernia of abdominal cavity & intestinal obstruction | K35–K46, K56 |
|  | Certain chronic liver diseases & cirrhosis | K70, K73, K74, K76 |
|  | Other diseases of digestive system (remaining K00–K93) | (see ranges above) |
|  | Diseases of the genitourinary system | N00–N39 |
|  | Hyperplasia of prostate | N40 |
|  | Pregnancy, childbirth & puerperium | O00–O99 |
|  | Congenital malformations, deformations & chromosomal abnormalities | Q00–Q99 |
|  | Other diseases (residual) |  |
| Symptoms, Signs & Ill-Defined Conditions | Symptoms, signs & ill-defined conditions | R00–R99 |
| COVID-19 | COVID-19, virus identified | U071 |
|  | COVID-19, virus not identified | U072 |
|  | Personal history of COVID-19 | U08, U089 |
|  | Post COVID-19 condition | U09, U099 |
|  | Multisystem inflammatory syndrome associated with COVID-19 | U10, U109 |
|  | immunization against COVID-19 | U11, U119 |
|  | COVID-19 vaccines causing adverse effects in therapeutic use | U12, U129 |
|  | Coronavirus infection, unspecified site | B342 |

## Weighted multiple cause of death

Part II of the death certificates had a maximum of 11 causes of death in Colombia and 7 in Brazil. In this study, we used the UC, as other diseases present in Part I are usually used to select the UC and do not correspond to causes of death. From Part II we used all diseases listed, but only one cause of death was accounted for when two or more in the certificate corresponded to the same group.

To formalize the weighting strategies, consider $w_{ic}$ represents the weight assigned to each cause of death reported in the certificate; $i$ is the index 1…n representing the decedents; $c$ is the index for 1 … $C_{i}$ causes of death reported for decedent $i$, where c=1 corresponds to the established underlying cause of death (part I of the certificate) and c>1 to the concomitant causes (part II of the certificate). As reported in the first column of Table S2, the traditional UC can be viewed as a special weighted MC whereby the underlying cause of death receives a weight of one and the concomitant causes a weight of zero. The MC approaches implemented in this study assign a non-zero weight to the concomitant causes of death reported in part II of the certificate. These two strategies are the most commonly applied in the literature^[[2]](#footnote-2),^^[[3]](#footnote-3)^

MC1 or half UC was the weighting strategy implemented in the main analyses. It maintains a higher relevance –i.e., weight– for the selected UCOD, while simultaneously distributing the weight among the concomitant causes (see second column of Table S2).

MC2 or equal weights was implemented as a sensitivity analysis and is reported in the third column of Table S2. This approach considers all causes reported in the certificate as equally important in the process of death and therefore divides the total weight equally among the reported causes.

Table S2. Weighting strategies

| Traditional (UC) | MC1: 50% UC | MC2: Equal weights |
| --- | --- | --- |
| $w_{ic}=\left\{ \begin{aligned} 1 c=1 \\ 0 c>1 \end{aligned} \right.$ | $w_{ic}=\left\{ \begin{aligned} 0.5 c=1 \\ \frac{0.5}{C_{i}} c>1 \end{aligned} \right.$ | $w_{ic}=\frac{1}{C_{i}}$ |

## Missing data

As explained in the main document, our data had very low percentages of death records with missing information for sex and age. Like all our analyses, the missing data were treated separately by country.

The variable sex was binary in both countries, with 0.02% and 0.04% missing in Colombia and Brazil, respectively. Age, however, was published as a categorical variable of age groups in Colombia (0.01% missing) and as years of age in Brazil, where there was a 0.24% of missingness.

To address this issue, we conducted single imputation using chained equations (in STATA, mi impute chained). The imputation is done conditionally on all other complete or imputed variables in the data.

Briefly, for the sex variable, a logistic regression is used to model the probability of each category in both countries. But age was treated differently. In Colombia, a multinomial logistic regression was used, and for Brazil, we used a predictive mean matching model. The latter was done with k=1, meaning the missing value is replaced with an observed value from the case with the closest predicted mean. Given the low proportions of missingness, we opted for just one imputation in both countries.

See the relevant section of the code below:

Colombia:

mi set wide

mi register imputed sex agegr

mi impute chained (logit)sex (ologit) agegr, add(1)

Brazil:

mi set wide

mi register imputed sex idade

mi impute chained (logit) sex (pmm, knn(1)) idade, add(1) burnin(5) noisily

## Age-specific mortality rates for Neoplasms. Colombia.


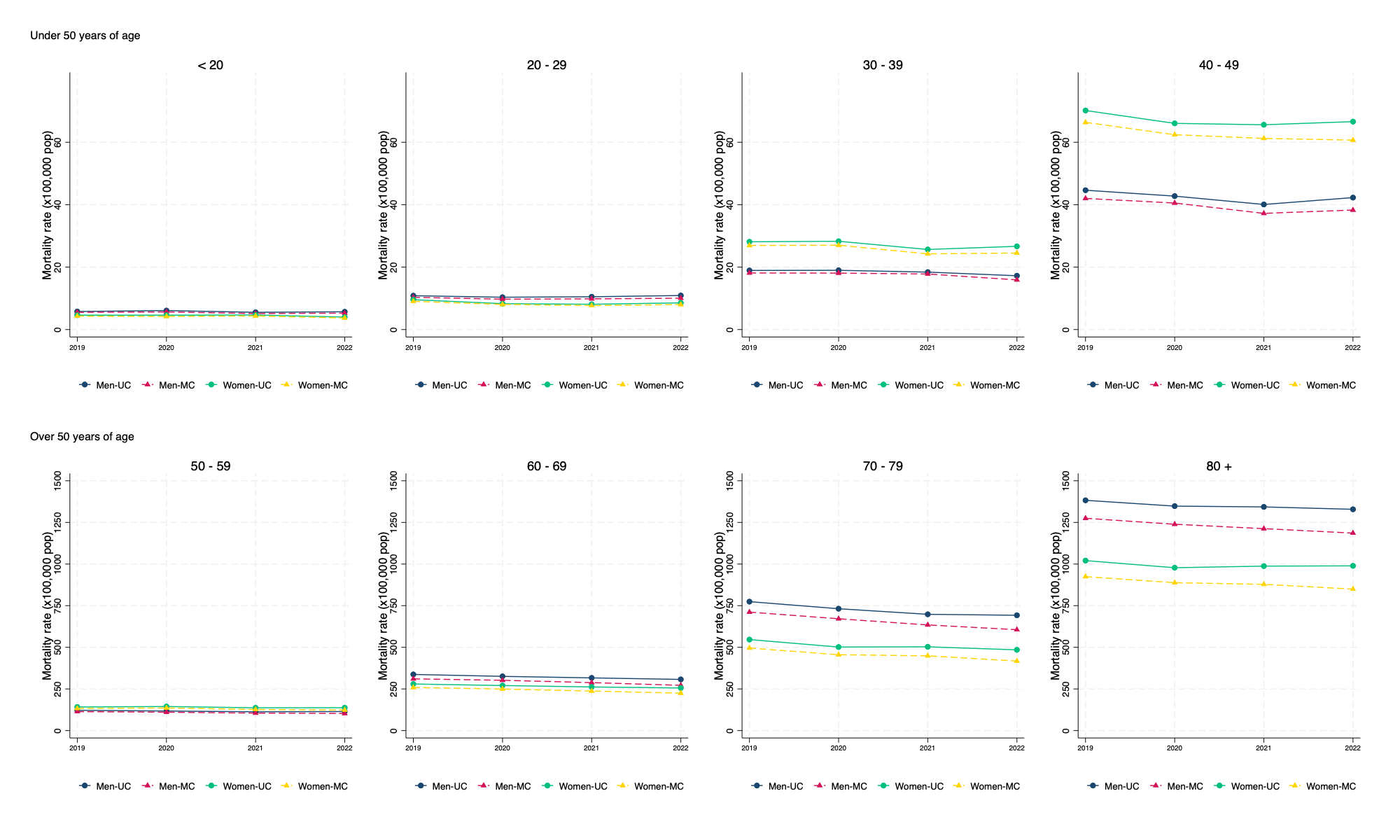


## Age-specific mortality rates for Circulatory System Diseases. Colombia.


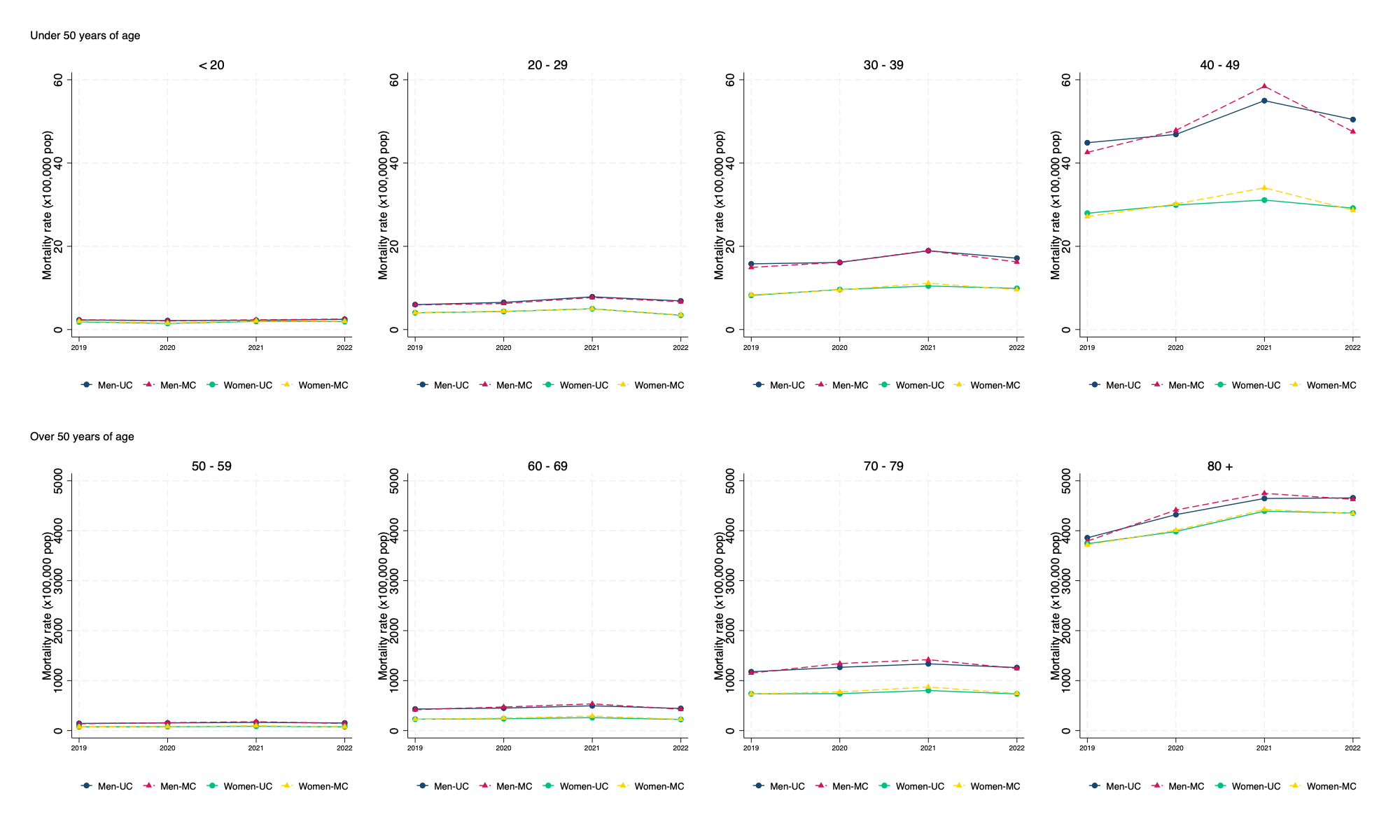


## Age-specific mortality rates for Communicable diseases. Colombia.


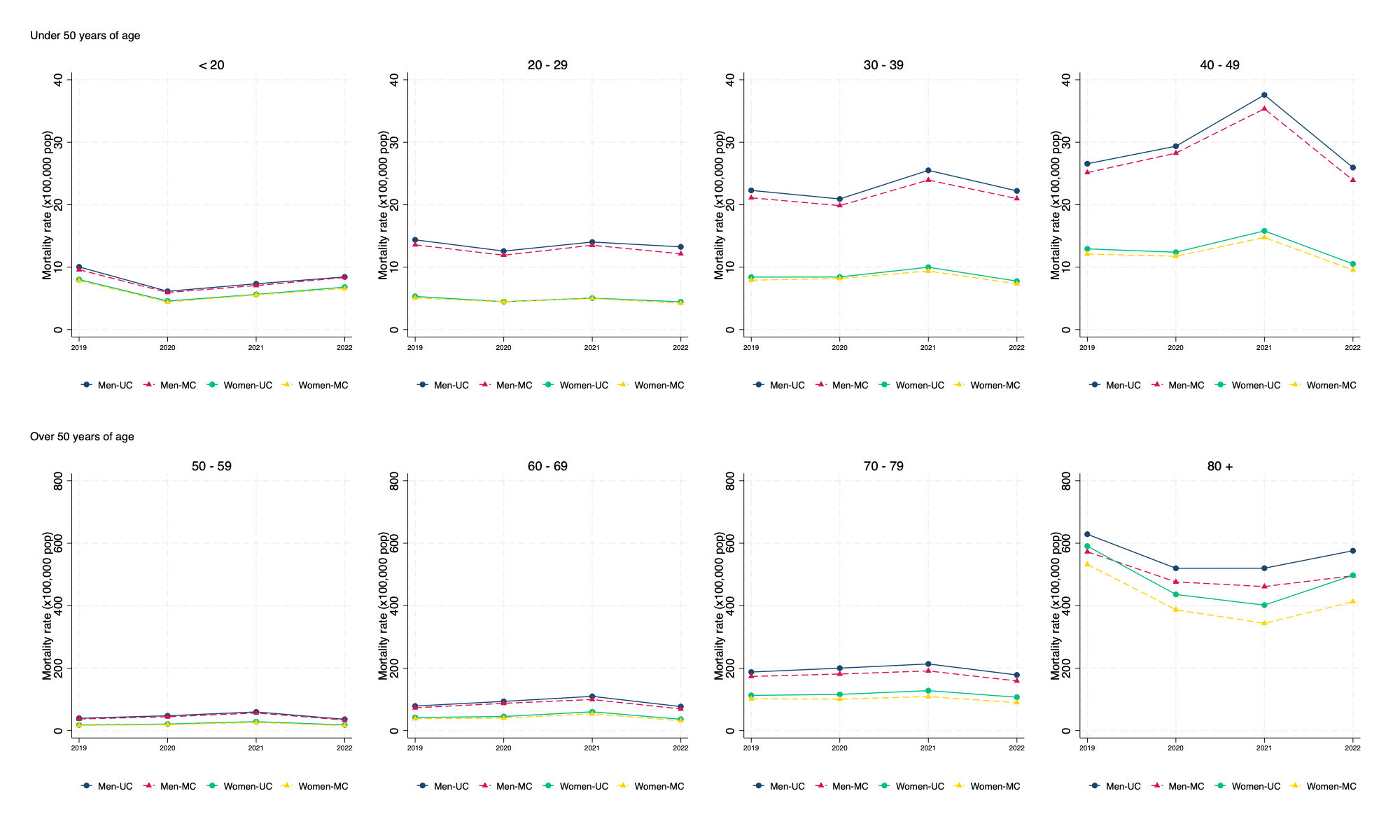


## Age-specific mortality rates for Diabetes Colombia.


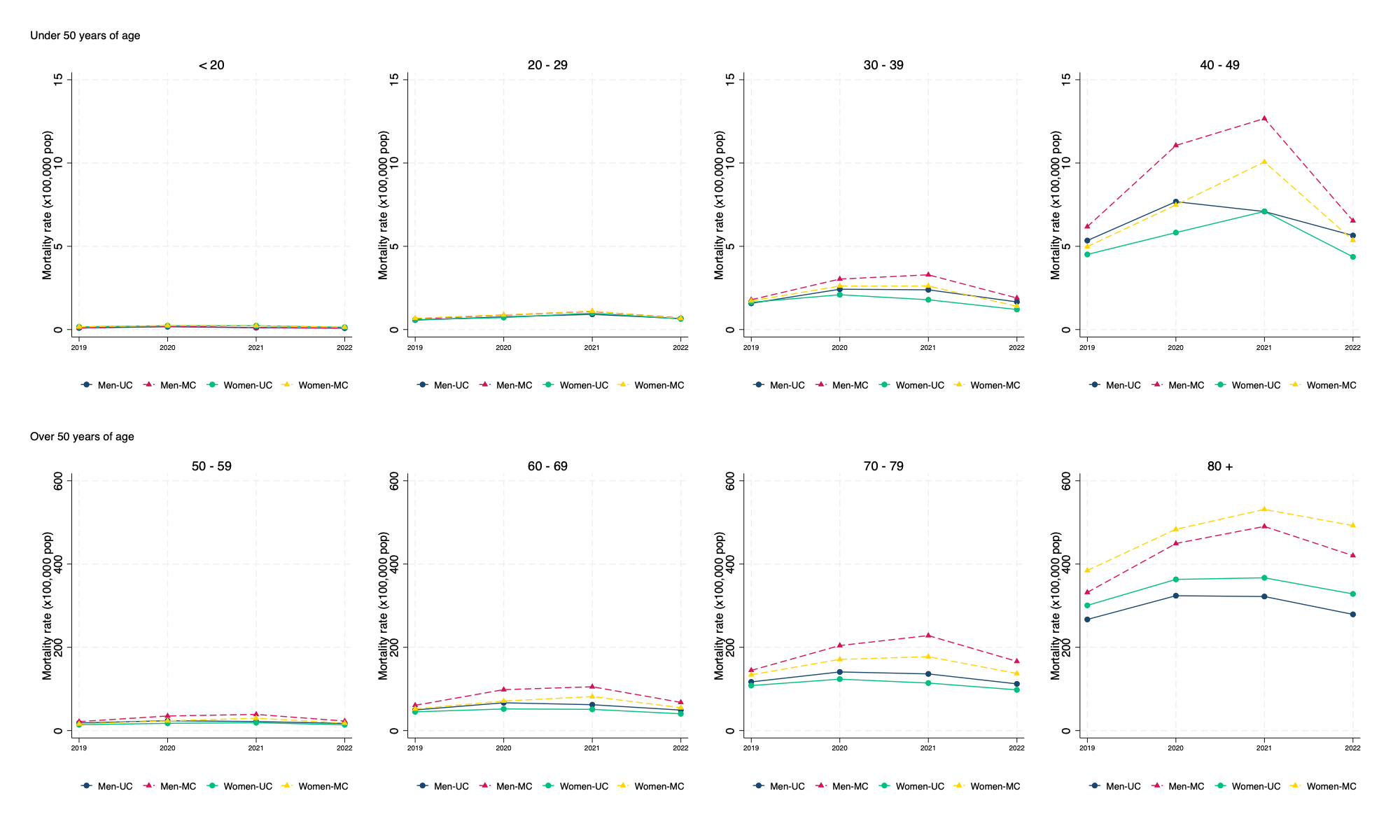


## Age-specific mortality rates for Neoplasms, Brazil.


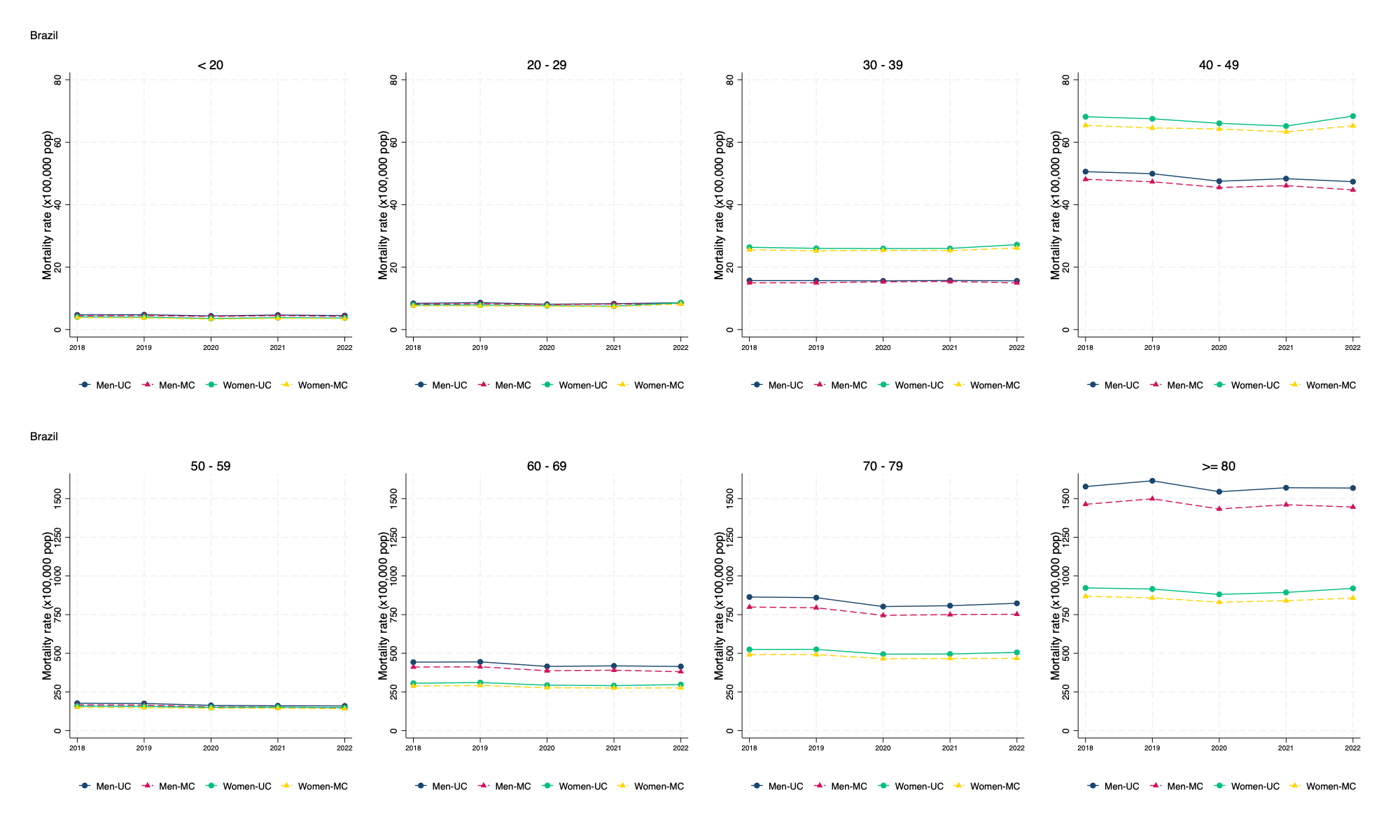


## Age-specific mortality rates for Circulatory System Diseases. Brazil.


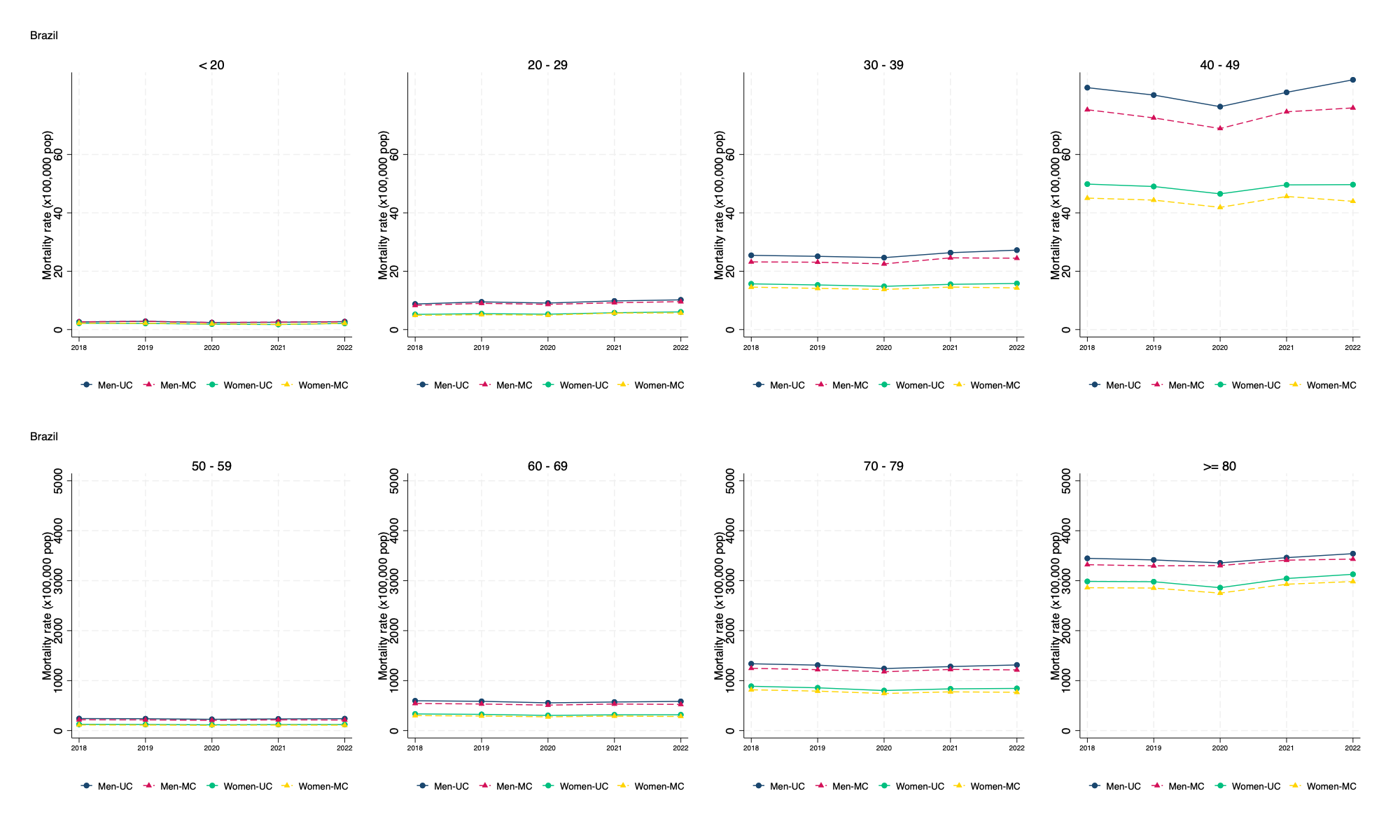


## Age-specific mortality rates for Communicable diseases. Brazil.


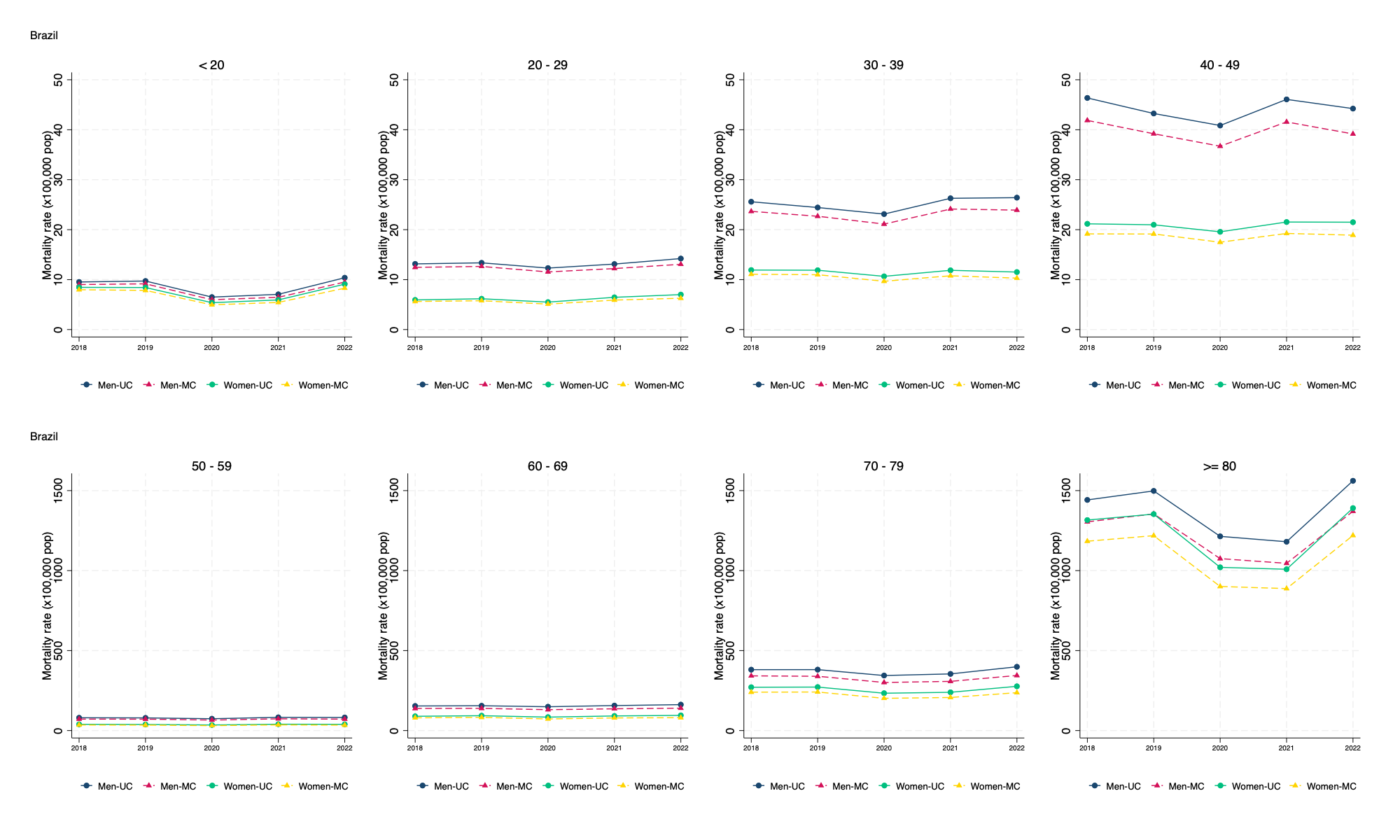


## Age-specific mortality rates for Diabetes. Brazil


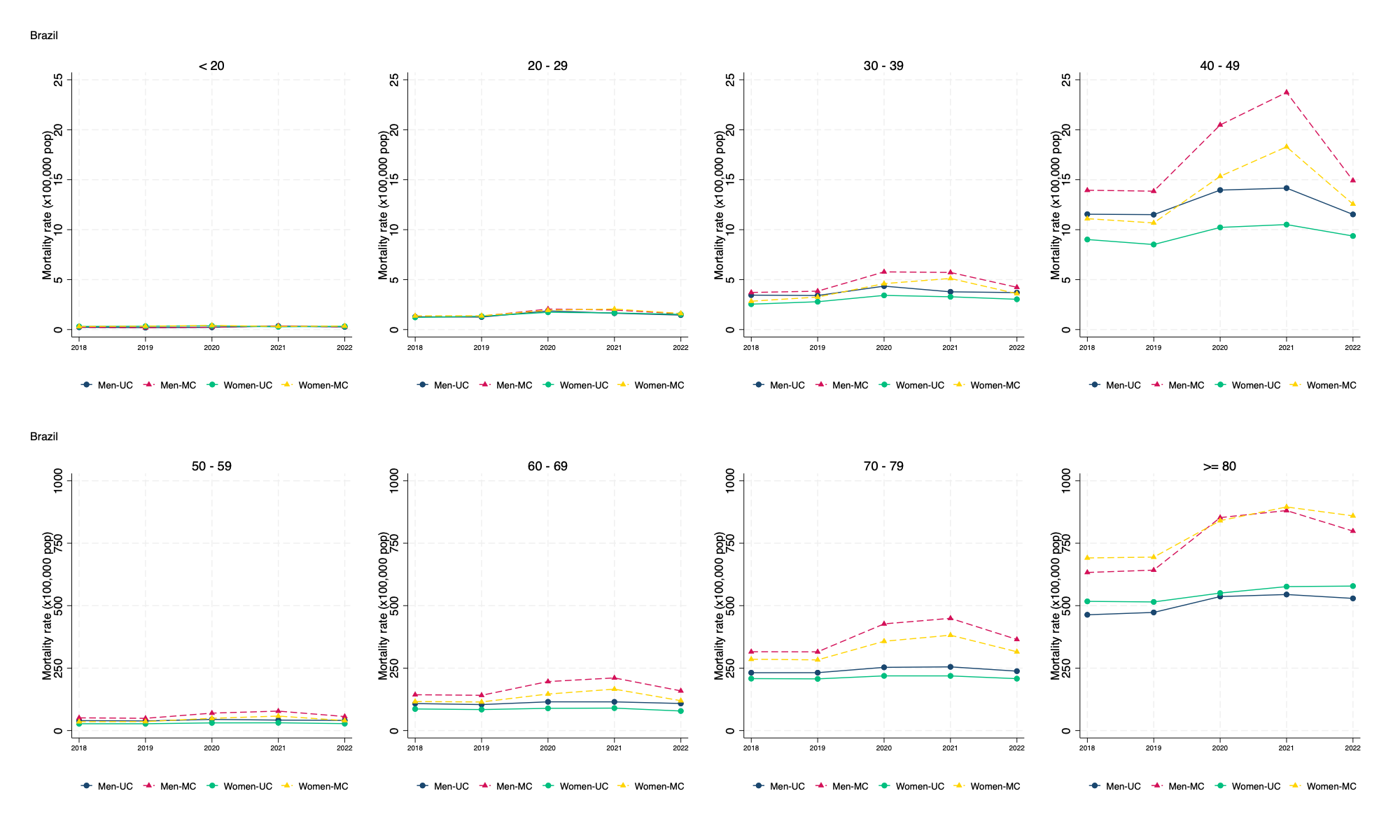


## Monthly variation in mortality, underlying and weighted multiple cause of death. Colombia.

1. Neoplasms


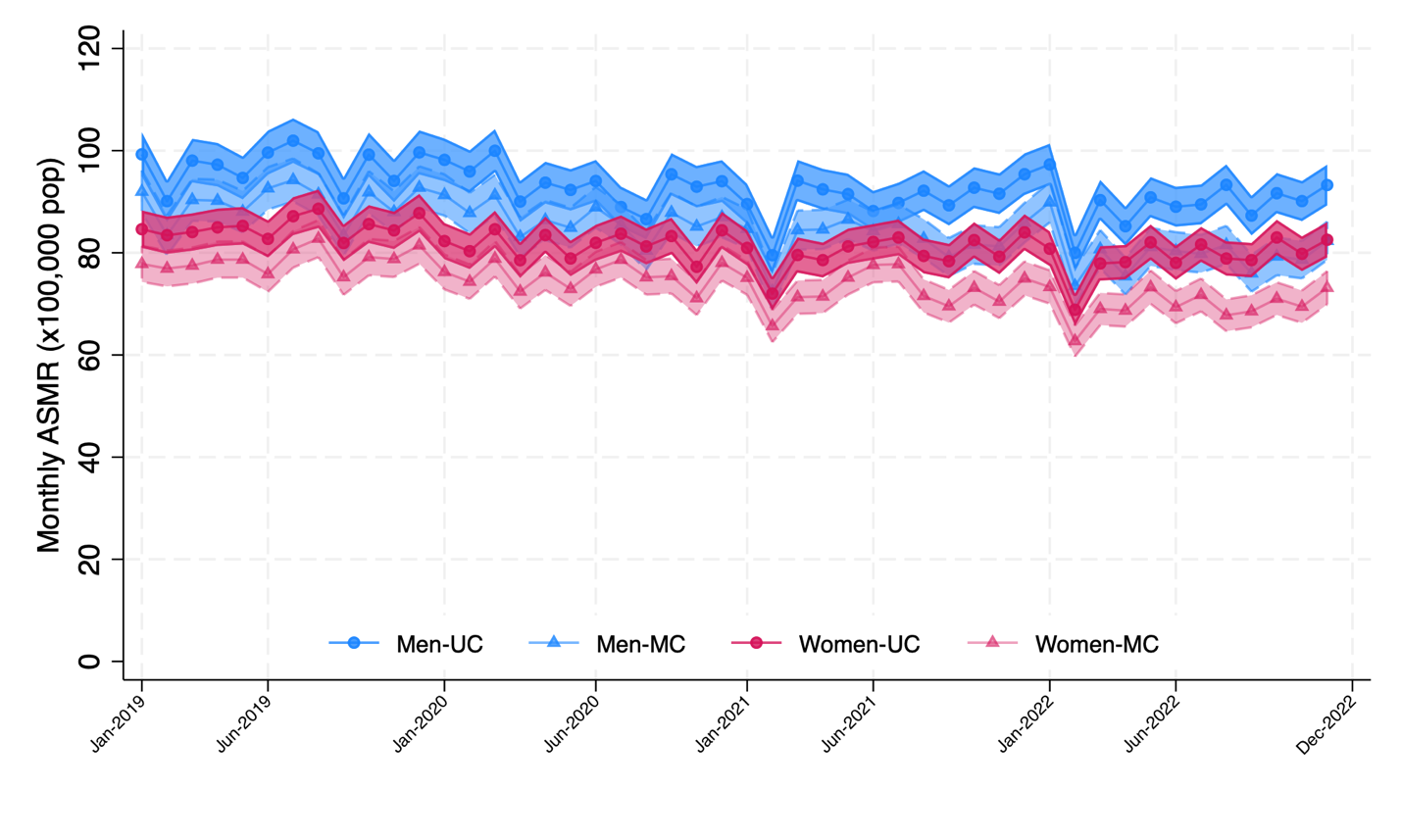


1. Circulatory system mortality


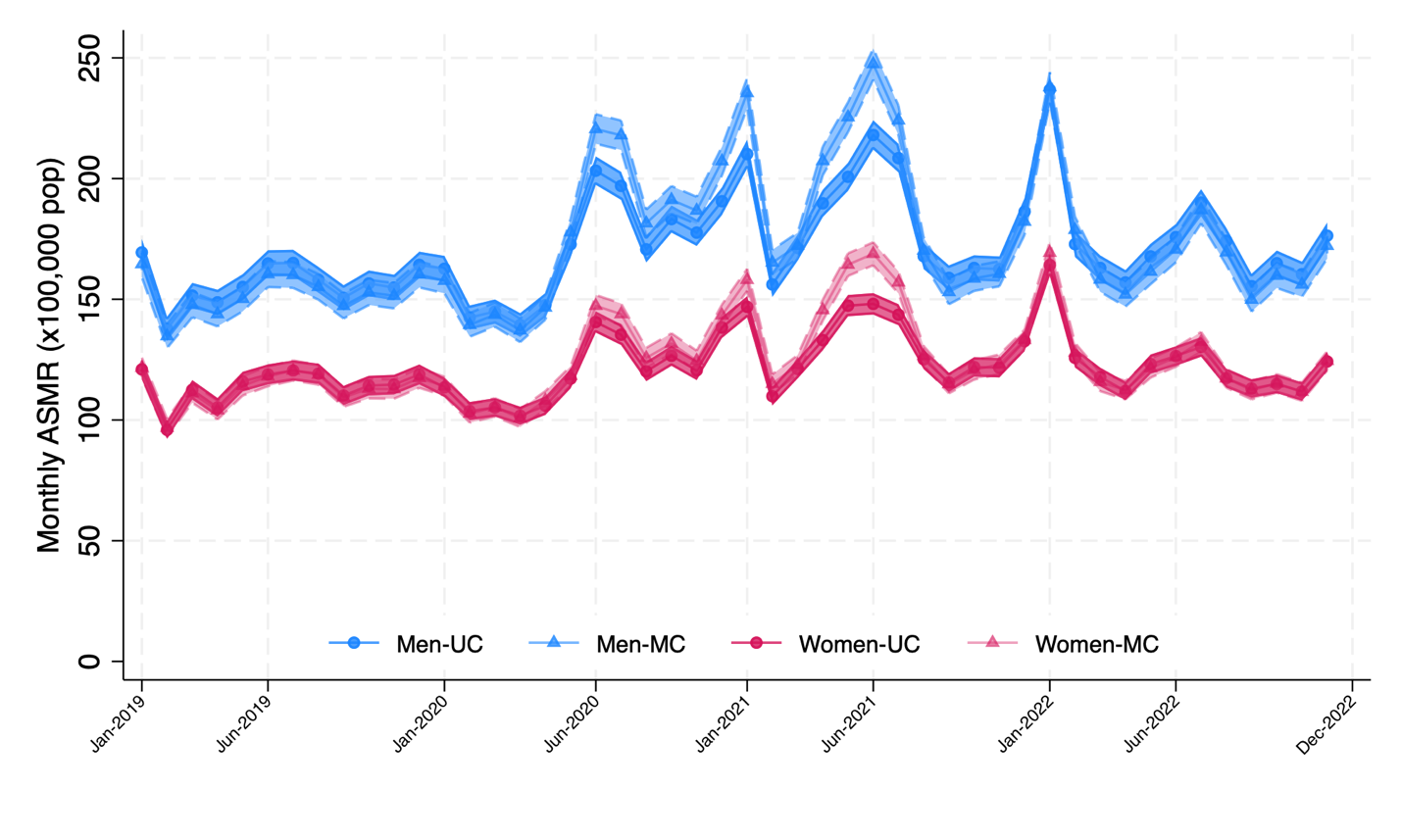


1. Communicable diseases mortality


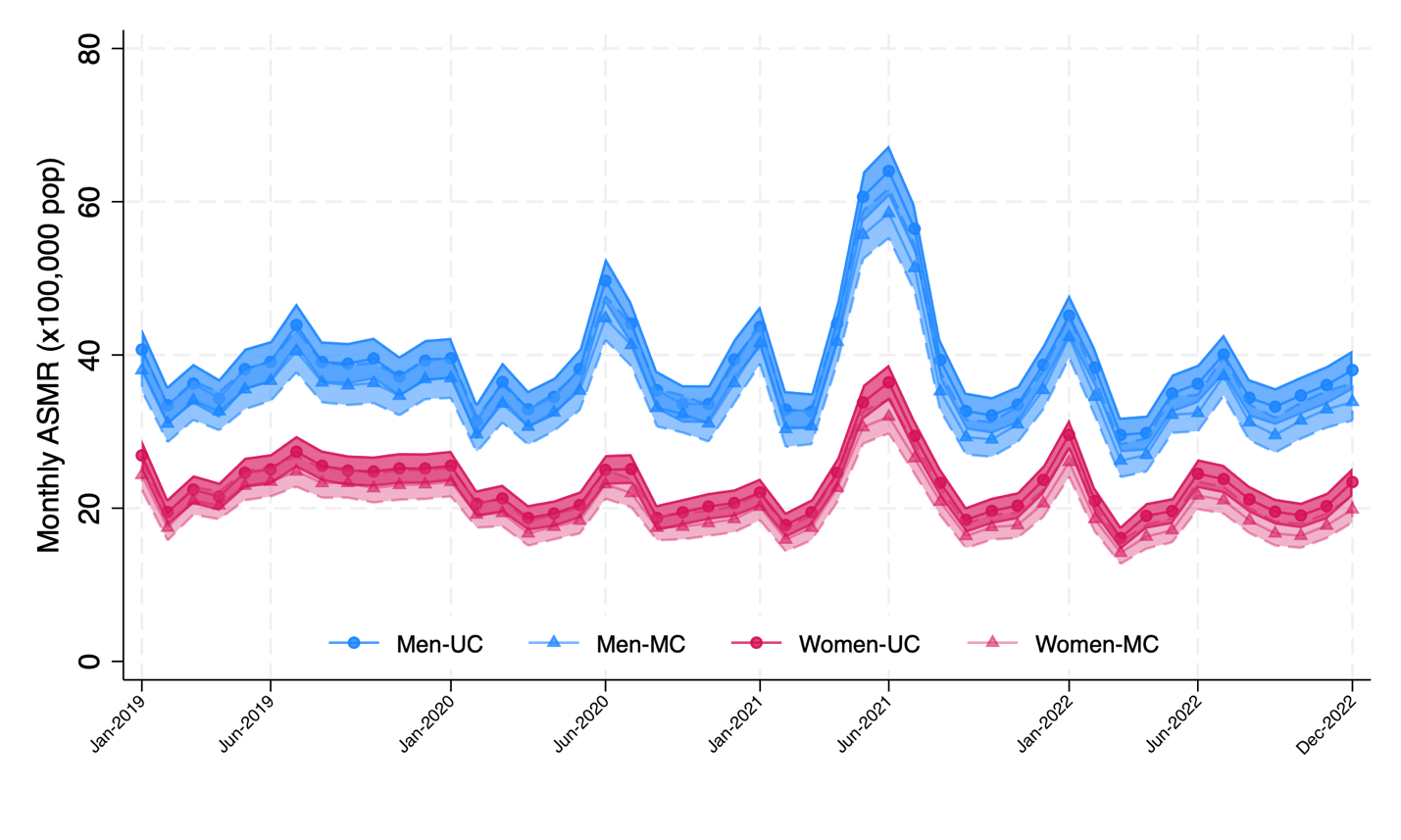


1. Diabetes Mellitus mortality


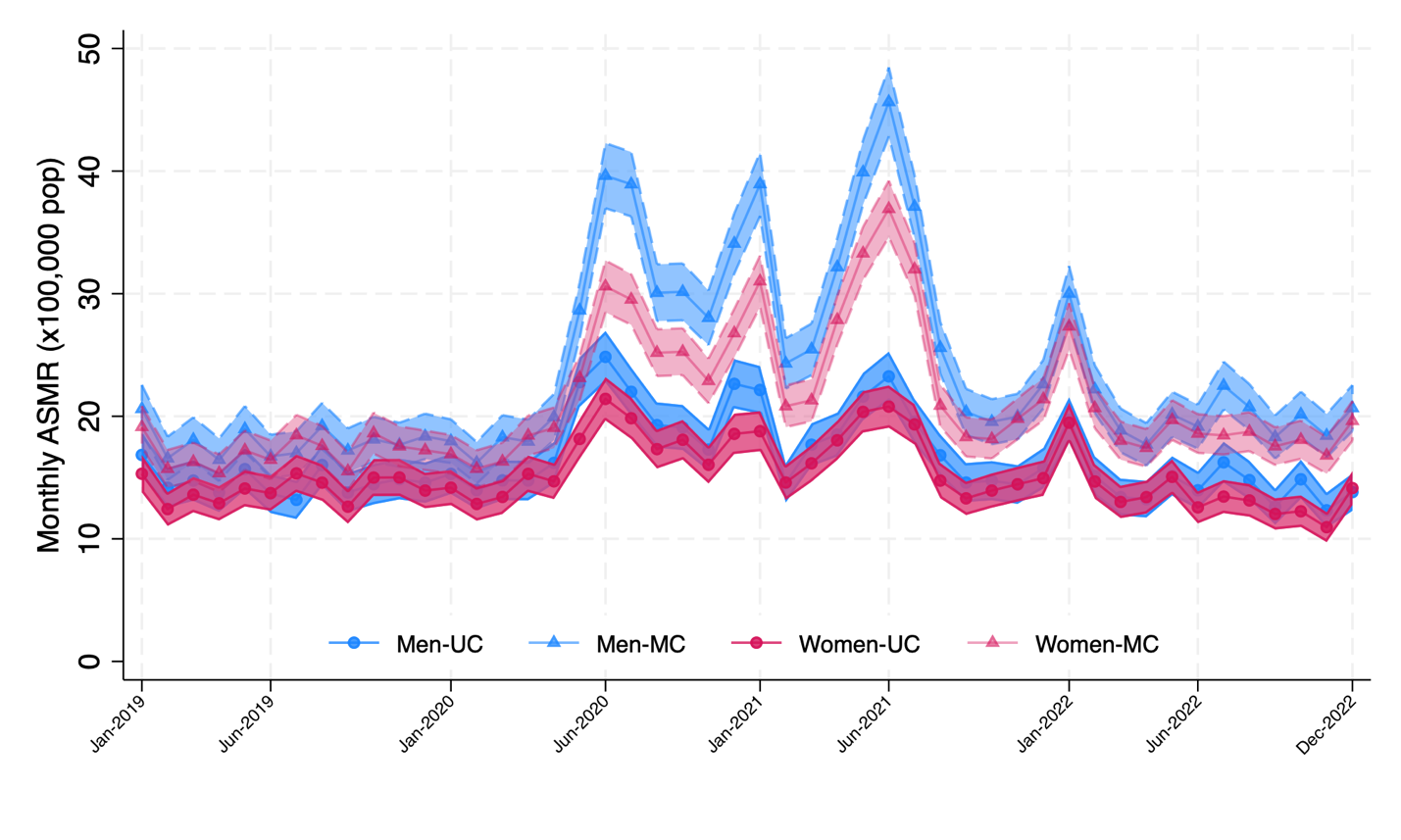


## Monthly variation in mortality, underlying and weighted multiple cause of death. Brazil.

1. Neoplasms


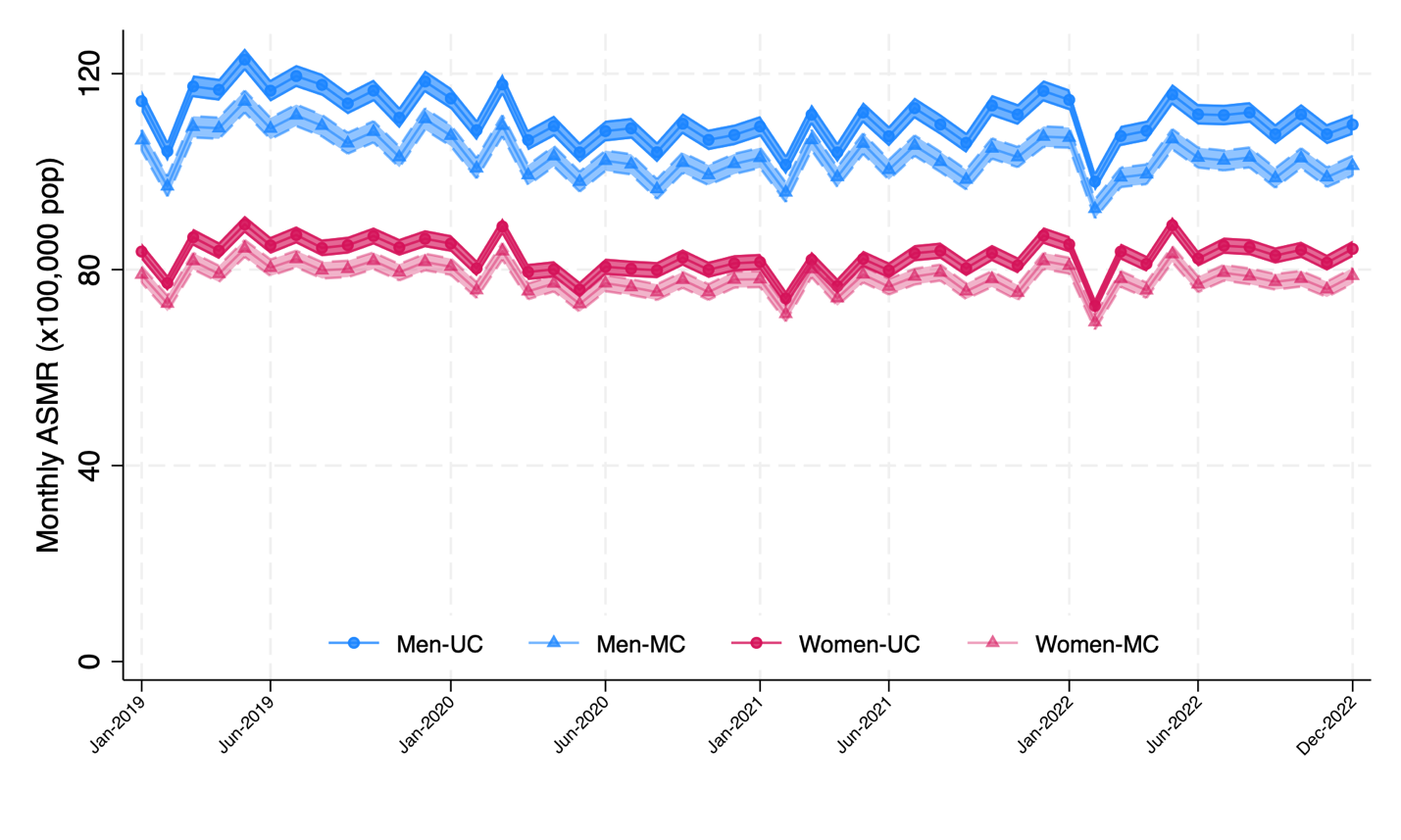


1. Circulatory system mortality


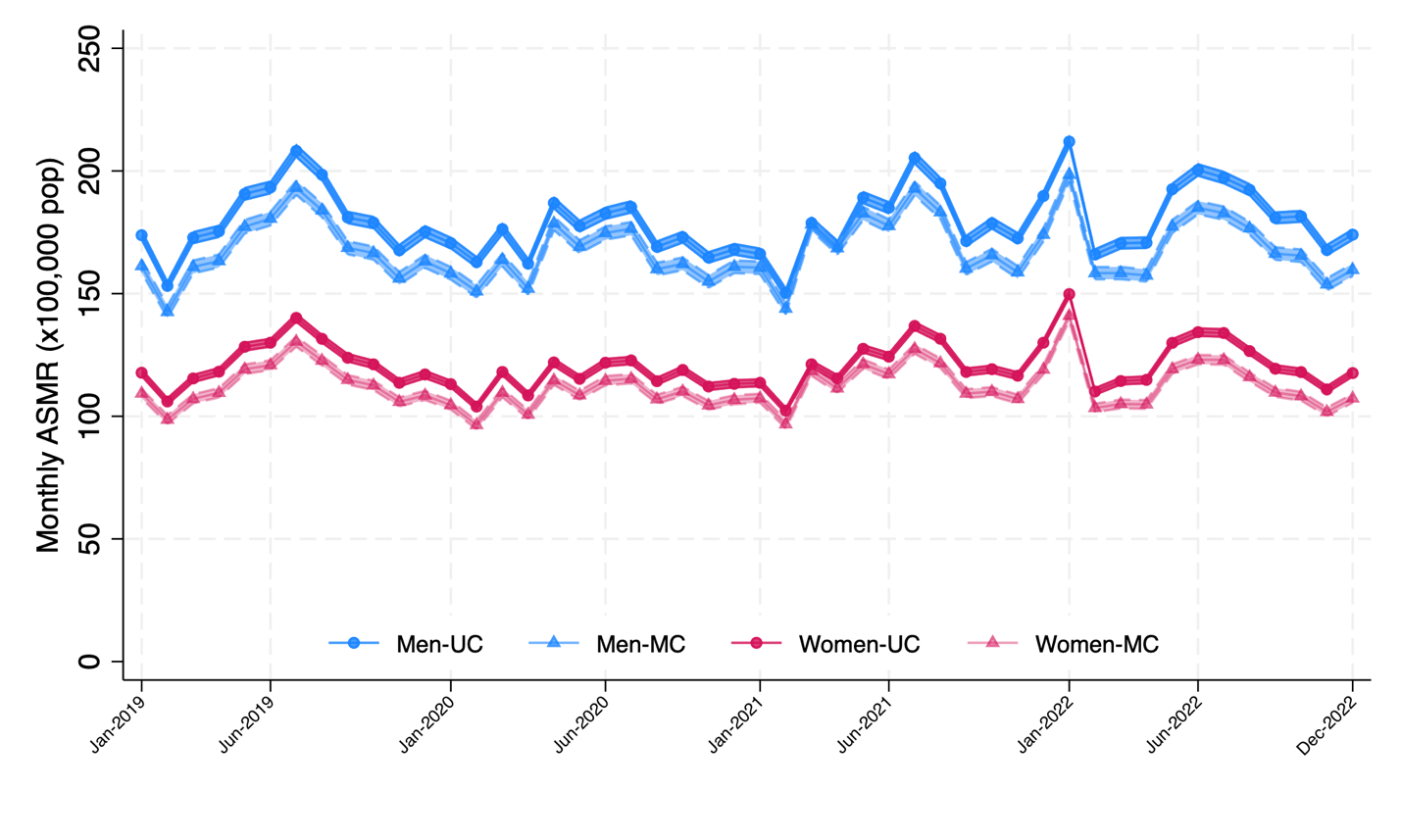


1. Communicable diseases mortality


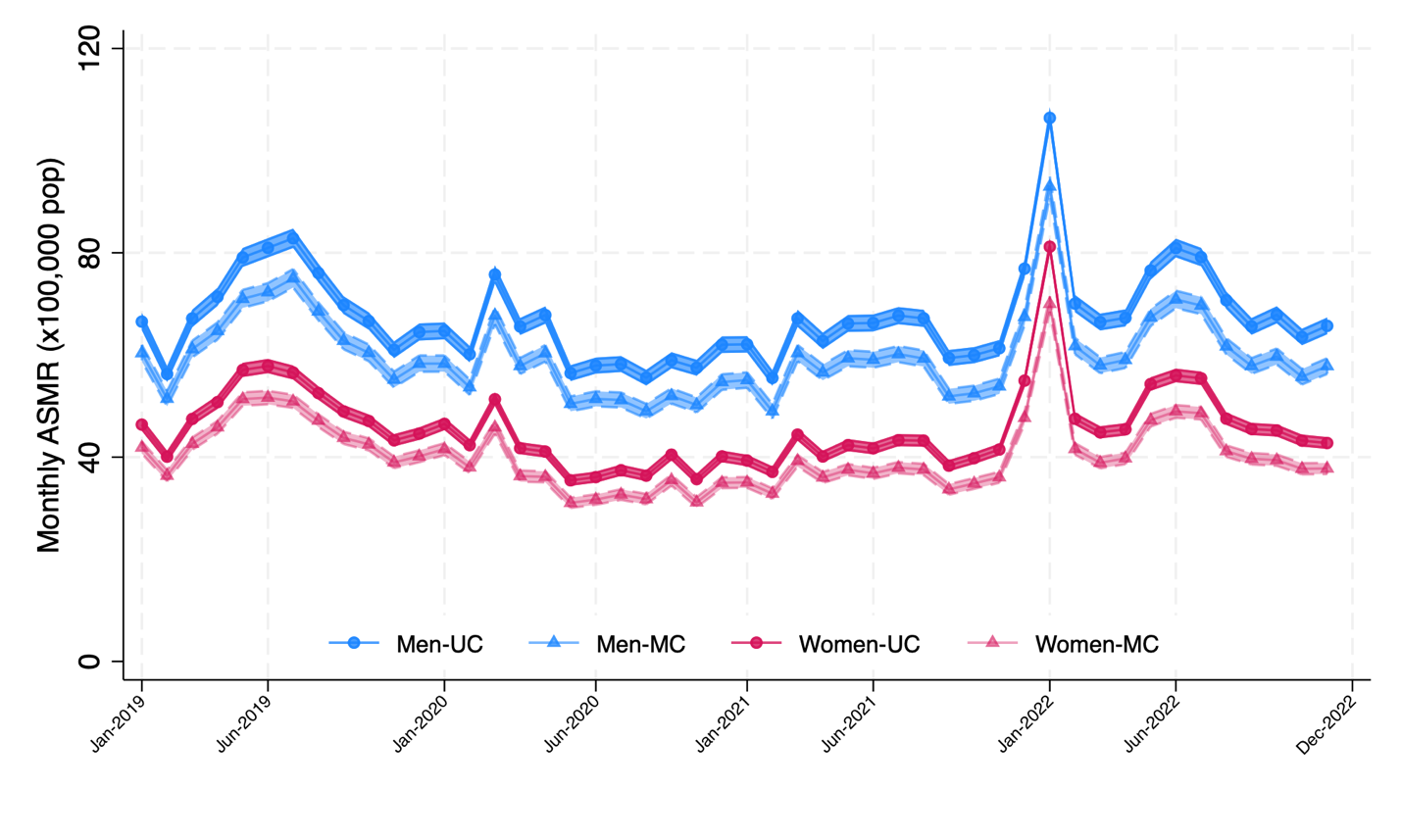


1. Diabetes Mellitus mortality


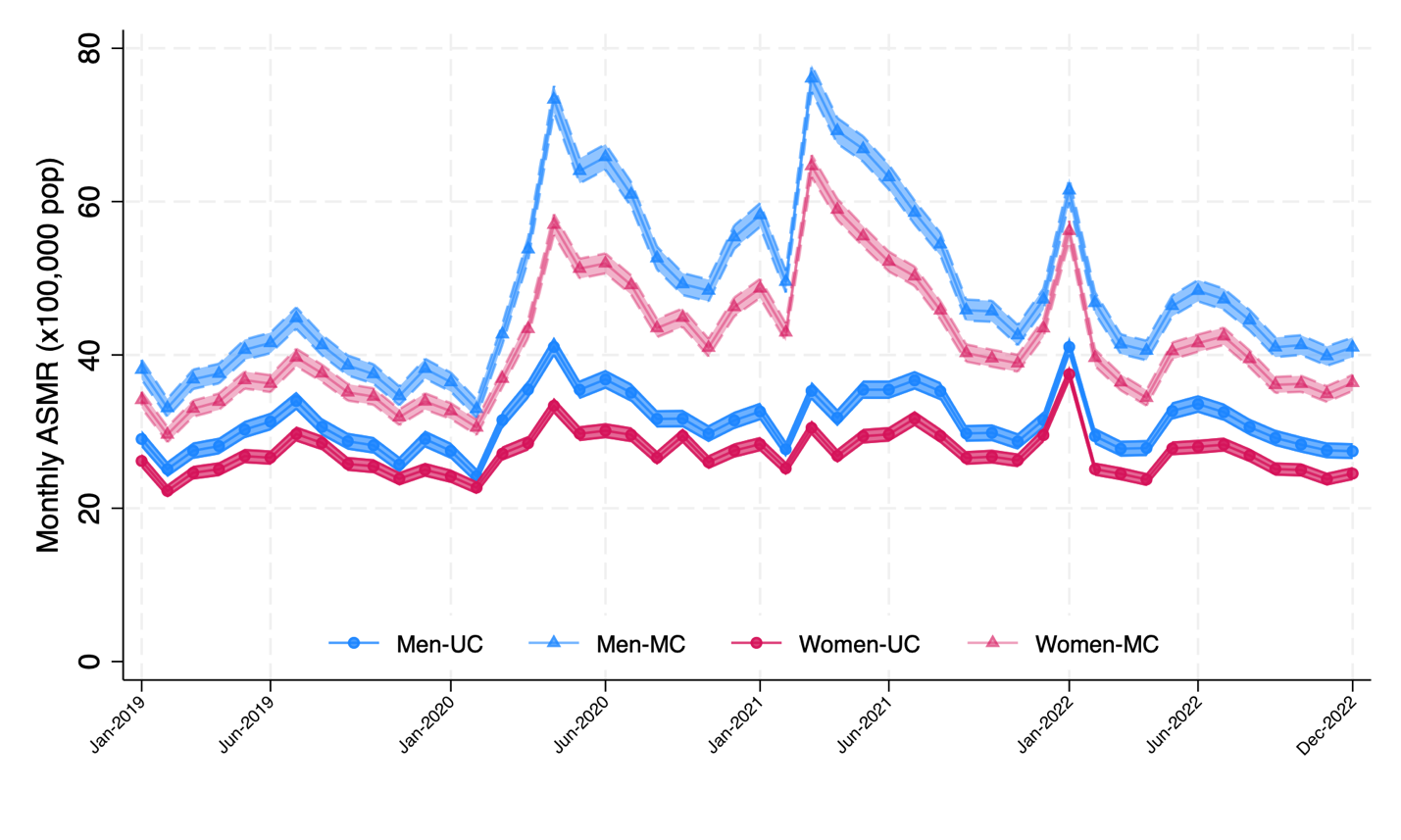


## Absolute and relative differences for MCOD versus UCOD mortality

Table S3. Absolute (AD) and relative differences (RD) for MCOD versus UCOD mortality. Colombia

|  | **Year** | **Sex** | **RD** | **AD** |
| --- | --- | --- | --- | --- |
| **Neoplasms** | 2019 | Men | 0.93 (0.91; 0.94) | -7.17 (-8.88; -5.46) |
|  | 2019 | Women | 0.92 (0.91; 0.94) | -6.48 (-7.96; -5.01) |
|  | 2020 | Men | 0.93 (0.91; 0.94) | -6.91 (-8.55; -5.27) |
|  | 2020 | Women | 0.92 (0.91; 0.94) | -6.19 (-7.61; -4.78) |
|  | 2021 | Men | 0.91 (0.90; 0.93) | -7.78 (-9.37; -6.19) |
|  | 2021 | Women | 0.91 (0.89; 0.93) | -7.31 (-8.69; -5.94) |
|  | 2022 | Men | 0.89 (0.87; 0.91) | -9.93 (-11.48; -8.37) |
|  | 2022 | Women | 0.88 (0.87; 0.90) | -9.39 (-10.73; -8.05) |
|  |  |  |  |  |
| **Circulatory system diseases (CVD)** | 2019 | Men | 0.97 (0.96; 0.99) | -4.07 (-6.25; -1.90) |
|  | 2019 | Women | 0.99 (0.98; 1.01) | -0.81 (-2.47; 0.86) |
|  | 2020 | Men | 1.04 (1.02; 1.05) | 6.25 (4.00; 8.49) |
|  | 2020 | Women | 1.03 (1.01; 1.04) | 3.03 (1.36; 4.71) |
|  | 2021 | Men | 1.05 (1.04; 1.06) | 9.05 (6.73; 11.36) |
|  | 2021 | Women | 1.05 (1.04; 1.07) | 6.91 (5.17; 8.65) |
|  | 2022 | Men | 0.98 (0.97; 0.99) | -3.45 (-5.65; -1.25) |
|  | 2022 | Women | 1.00 (0.99; 1.02) | 0.40 (-1.24; 2.04) |
|  |  |  |  |  |
| **Diabetes Mellitus** | 2019 | Men | 1.22 (1.17; 1.28) | 3.25 (2.54; 3.96) |
|  | 2019 | Women | 1.21 (1.17; 1.27) | 3.02 (2.39; 3.65) |
|  | 2020 | Men | 1.44 (1.38; 1.49) | 8.09 (7.27; 8.90) |
|  | 2020 | Women | 1.35 (1.30; 1.40) | 5.81 (5.12; 6.49) |
|  | 2021 | Men | 1.64 (1.59; 1.70) | 11.47 (10.64; 12.29) |
|  | 2021 | Women | 1.51 (1.46; 1.57) | 8.52 (7.82; 9.22) |
|  | 2022 | Men | 1.42 (1.36; 1.48) | 6.14 (5.43; 6.85) |
|  | 2022 | Women | 1.40 (1.35; 1.46) | 5.54 (4.93; 6.15) |
|  |  |  |  |  |
| **Communicable**  **diseases (CD)** | 2019 | Men | 0.93 (0.90; 0.96) | -2.65 (-3.72; -1.57) |
|  | 2019 | Women | 0.92 (0.89; 0.95) | -1.99 (-2.77; -1.20) |
|  | 2020 | Men | 0.93 (0.90; 0.96) | -2.62 (-3.66; -1.58) |
|  | 2020 | Women | 0.91 (0.87; 0.94) | -2.01 (-2.72; -1.29) |
|  | 2021 | Men | 0.92 (0.89; 0.94) | -3.43 (-4.53; -2.34) |
|  | 2021 | Women | 0.89 (0.86; 0.92) | -2.57 (-3.32; -1.81) |
|  | 2022 | Men | 0.91 (0.88; 0.93) | -3.34 (-4.34; -2.35) |
|  | 2022 | Women | 0.87 (0.84; 0.90) | -2.77 (-3.47; -2.07) |

Table S4. Absolute (AD) and relative differences (RD) for MCOD versus UCOD mortality. Brazil

|  | **Year** | **Sex** | **RD** | **AD** |
| --- | --- | --- | --- | --- |
| **Neoplasms** | 2019 | Men | 0.93 (0.92; 0.94) | -8.01 (-8.91; -7.11) |
|  | 2019 | Women | 0.94 (0.94; 0.95) | -4.78 (-5.47; -4.08) |
|  | 2020 | Men | 0.93 (0.93; 0.94) | -7.09 (-7.95; -6.23) |
|  | 2020 | Women | 0.95 (0.94; 0.96) | -4.01 (-4.68; -3.33) |
|  | 2021 | Men | 0.94 (0.93; 0.94) | -7.09 (-7.94; -6.24) |
|  | 2021 | Women | 0.95 (0.94; 0.96) | -3.97 (-4.64; -3.30) |
|  | 2022 | Men | 0.92 (0.91; 0.93) | -8.52 (-9.36; -7.68) |
|  | 2022 | Women | 0.94 (0.93; 0.94) | -5.30 (-5.96; -4.63) |
|  |  |  |  |  |
| **Circulatory system diseases (CVD)** | 2019 | Men | 0.93 (0.92; 0.94) | -12.60 (-13.73; -11.48) |
|  | 2019 | Women | 0.93 (0.92; 0.94) | -8.63 (-9.44; -7.82) |
|  | 2020 | Men | 0.94 (0.94; 0.95) | -9.79 (-10.88; -8.70) |
|  | 2020 | Women | 0.93 (0.93; 0.94) | -7.61 (-8.39; -6.84) |
|  | 2021 | Men | 0.95 (0.95; 0.96) | -8.79 (-9.89; -7.69) |
|  | 2021 | Women | 0.94 (0.93; 0.94) | -7.48 (-8.27; -6.70) |
|  | 2022 | Men | 0.92 (0.92; 0.93) | -13.86 (-14.96; -12.77) |
|  | 2022 | Women | 0.92 (0.91; 0.93) | -9.77 (-10.55; -9.00) |
|  |  |  |  |  |
| **Diabetes Mellitus** | 2019 | Men | 1.33 (1.31; 1.35) | 9.61 (9.11; 10.10) |
|  | 2019 | Women | 1.34 (1.33; 1.36) | 8.87 (8.46; 9.28) |
|  | 2020 | Men | 1.62 (1.60; 1.64) | 20.33 (19.78; 20.88) |
|  | 2020 | Women | 1.58 (1.56; 1.60) | 16.13 (15.69; 16.57) |
|  | 2021 | Men | 1.74 (1.71; 1.76) | 23.95 (23.40; 24.50) |
|  | 2021 | Women | 1.71 (1.69; 1.73) | 20.08 (19.63; 20.54) |
|  | 2022 | Men | 1.47 (1.45; 1.49) | 14.33 (13.83; 14.84) |
|  | 2022 | Women | 1.48 (1.46; 1.50) | 12.82 (12.41; 13.23) |
|  |  |  |  |  |
| **Communicable**  **diseases (CD)** | 2019 | Men | 0.90 (0.89; 0.91) | -6.76 (-7.46; -6.06) |
|  | 2019 | Women | 0.90 (0.89; 0.91) | -4.96 (-5.48; -4.45) |
|  | 2020 | Men | 0.89 (0.88; 0.90) | -6.99 (-7.63; -6.35) |
|  | 2020 | Women | 0.88 (0.87; 0.89) | -4.84 (-5.30; -4.38) |
|  | 2021 | Men | 0.89 (0.88; 0.90) | -7.32 (-7.97; -6.67) |
|  | 2021 | Women | 0.88 (0.87; 0.89) | -5.07 (-5.53; -4.60) |
|  | 2022 | Men | 0.88 (0.87; 0.89) | -8.99 (-9.68; -8.30) |
|  | 2022 | Women | 0.87 (0.86; 0.88) | -6.52 (-7.03; -6.02) |

## Sensitivity analysis: alternative weighting scheme

##

Figure S16.1. Absolute differences (AD) for weighted MCOD versus UCOD mortality rates for neoplasms, circulatory system diseases, infectious diseases, and diabetes mellitus by sex


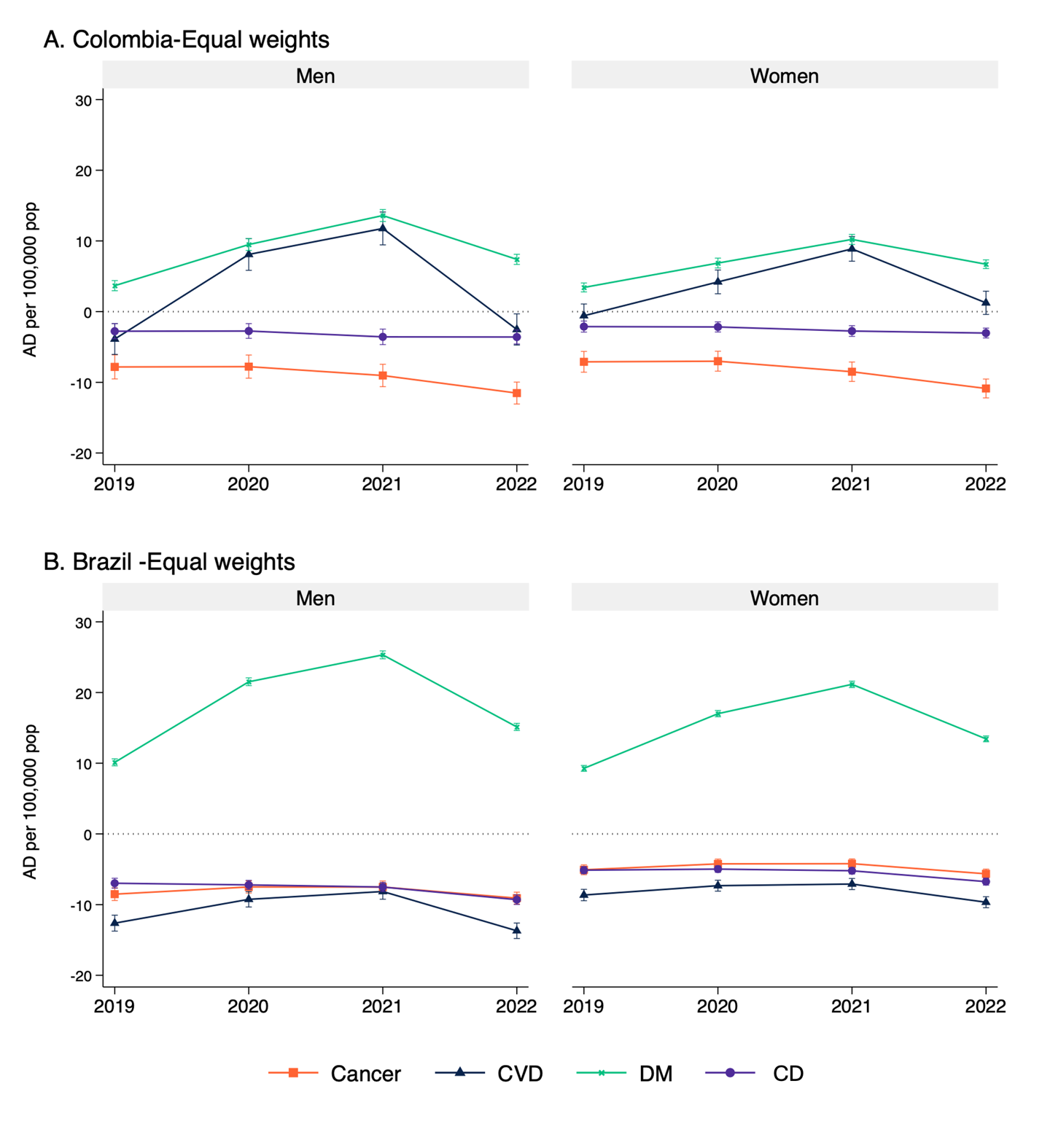


Figure S16.2. Relative differences (RD) for weighted MCOD versus UCOD mortality rates for neoplasms, circulatory system diseases, infectious diseases, and diabetes mellitus by sex


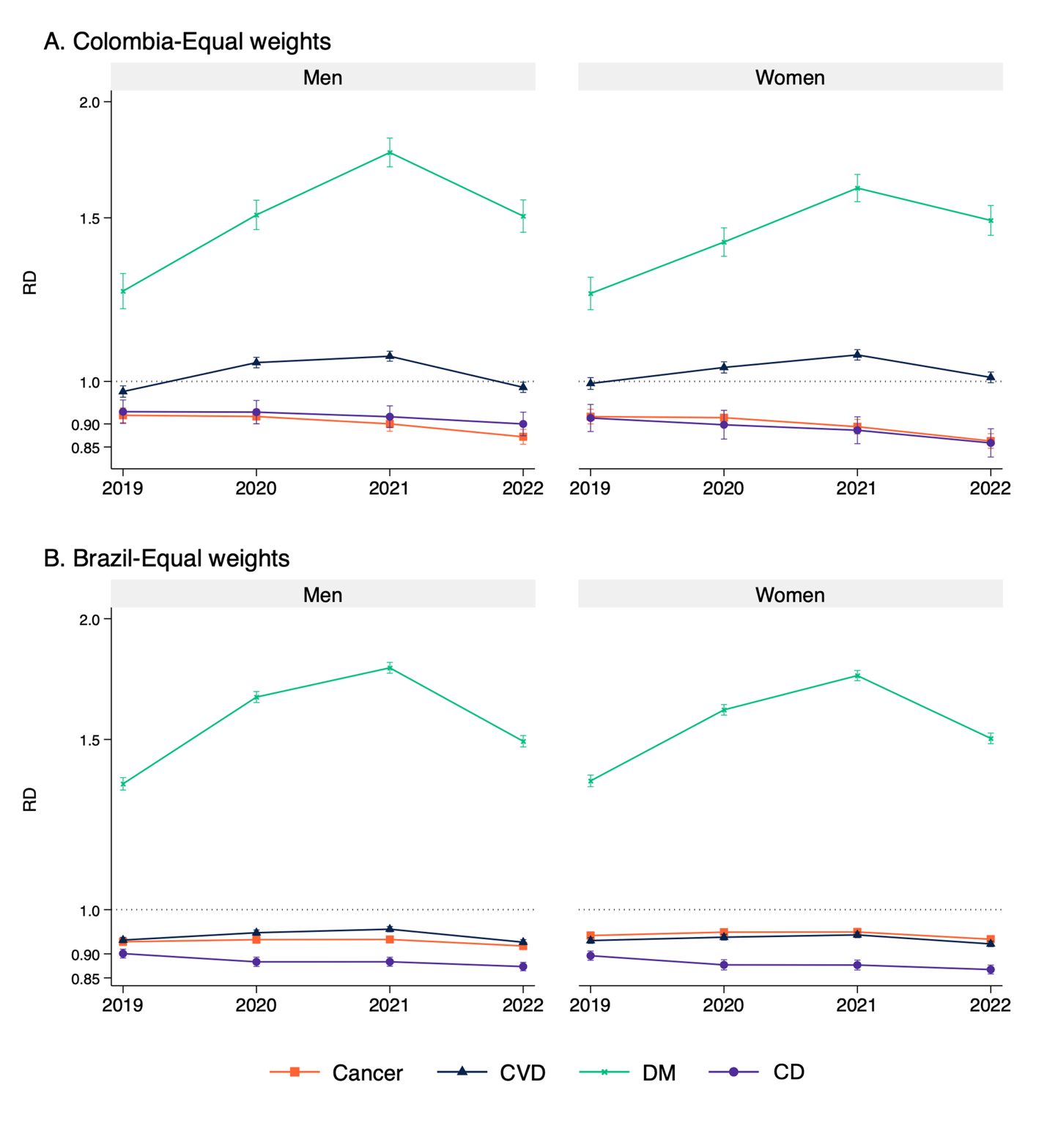


## Number of causes of death listed in Part II of the death certificate per country, sex and age.


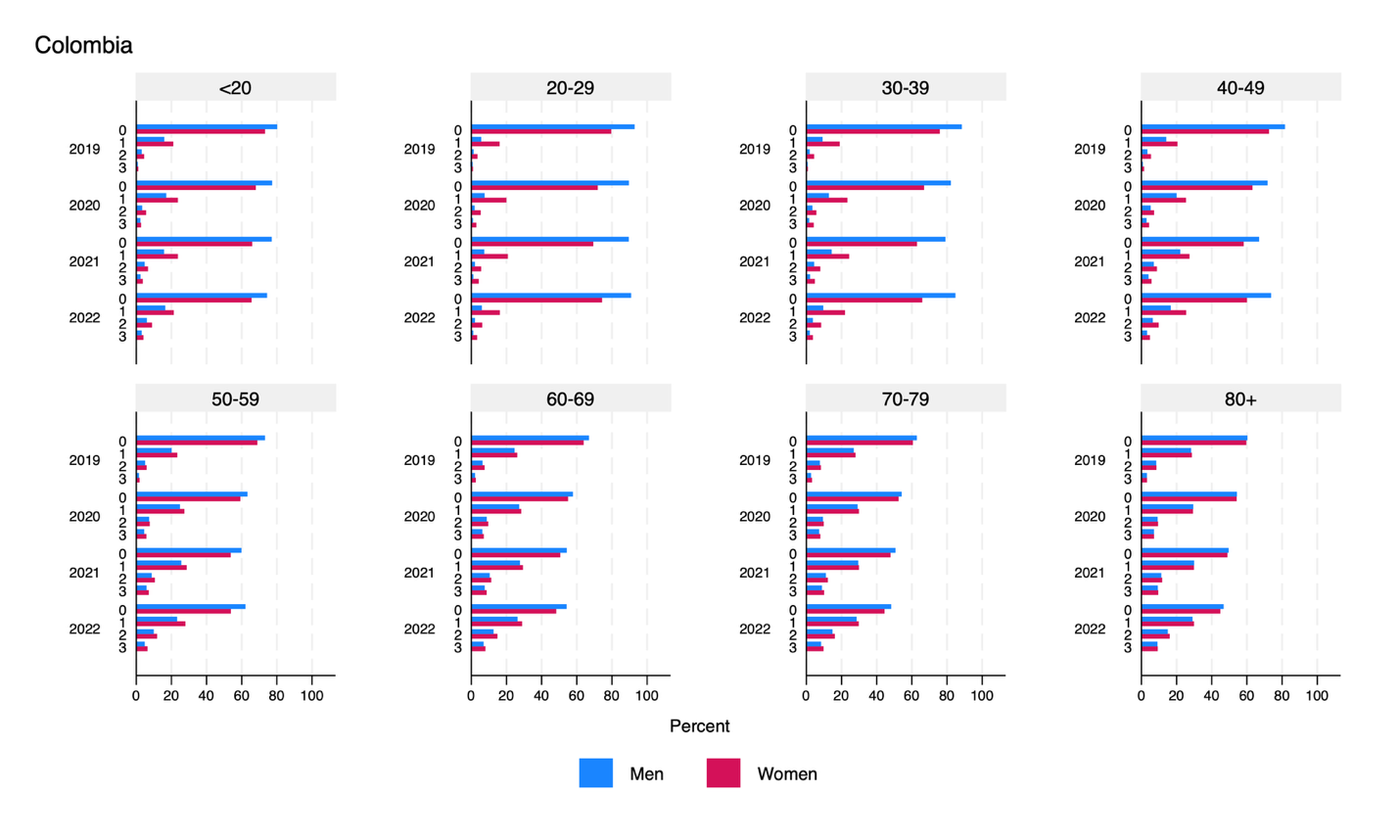


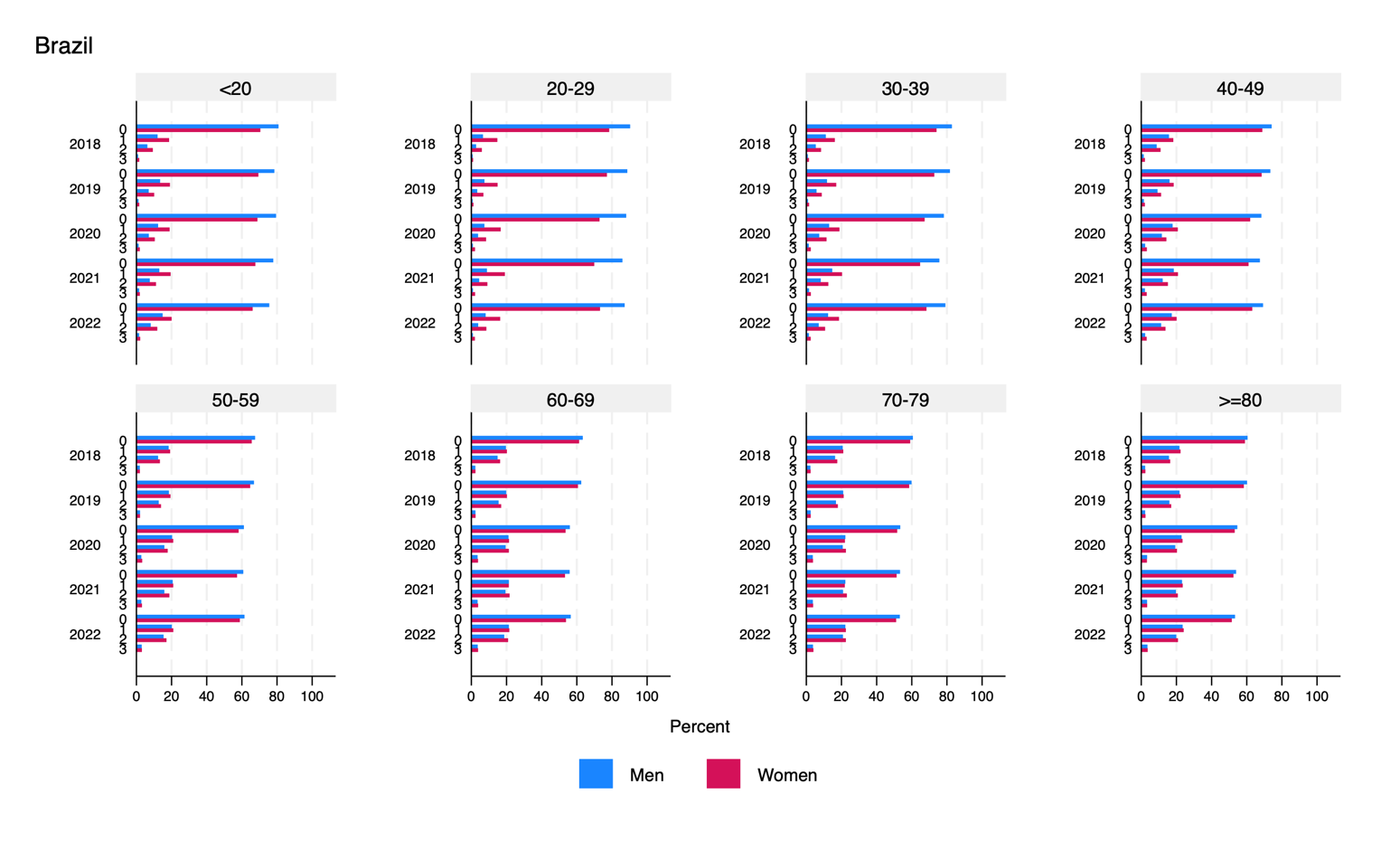


The bars represent the proportion of certificates with 0,1,2 and 3 or more causes reported in part II of the death certificates.

## Analysis script

1. **Weights**: This should be done after the data is clean and the ICD-10 codes in part 2 of the certificates are already labeled in groups according to the cause of death list selected.

**# Records of each cause group, either as UCOD or in part 2

** to identify if there are any of the causes in group 200 in part 2

//gen variables

foreach x in 201 202 203 204 205 206 207 208 211 212 213 214 215 209 210{

gen anyL`x'_p2= 0

}

// check part2 – Part 2 of the certificate can have up to 11 fields

forval i = 1(1)11 {

foreach x in 201 202 203 204 205 206 207 208 211 212 213 214 215 {

replace anyL`x'_p2 = 1 if L`x'_4_P2`i' == 1 | L`x'_3_P2`i' == 1

}

}

// to identify if they are UCOD or in part 2

forval x = 201(1)215 {

gen anyL`x'= 0

replace anyL`x' = 1 if L`x'==1 | anyL`x'_p2==1 // if in one or the other

}

// Indicator of UC or Contributing

forval x = 201(1)215 {

gen L`x'_type=.

replace L`x'_type=1 if L`x'==1 //underlying

replace L`x'_type=0 if L`x'!=1 & anyL`x'_p2==1 // contributing

}

**# Total Records (UCOD + part2). After creating all the groups (100, 200, etc), we create a records variable that is necessary for the weights

**********************************************************

gen records= anycov + anyL201 + anyL202 + anyL203 + anyL204 + anyL205 + anyL206 + anyL207 + anyL208 + anyL209 +… // all groups of causes of death

ta records, m

**# Weights: this should be repeated for each group of diseases in the cause of death list

**********************************************************

*** For L200 neoplasms

// gen vars

foreach x in 101 102 103 104 105 106 107 108 109 406 {

//double weight

gen L`x'wt_double= 0

replace L`x'wt_double= (2*[1 / (records+ 1)]) if L`x'_type==1

replace L`x'wt_double= [1 / (records+ 1)] if L`x'_type==0

replace L`x'wt_double= 0 if L`x'_type==.

// half weight

gen L`x'wt_50 =.

replace L`x'wt_50= (0.5/(records-1)) if L`x'_type==0 // half of the weight in contributing causes equally, -1 to not count UC if multiple records

replace L`x'wt_50= 0.5 if L`x'_type==1 // half of the weight to the ucod

replace L`x'wt_50= 1 if L`x'==1 & records==1 // all the weight to the ucod if only 1 record

replace L`x'wt_50= 0 if L`x'_type==.

// all weights divided equally

gen L`x'wt_eq =0

replace L`x'wt_eq= 1/records if anyL`x'==1

}

*** For COVID-19, weights are created separately

// double weight to covid as UC

gen covwt_double =.

replace covwt_double= (2*[1 / (records+ 1)]) if cov_type==1

replace covwt_double= [1 / (records+ 1)] if cov_type==0

replace covwt_double= 0 if cov_type==.

ta covwt_double ,m

//half of the weight to the covid UC, the rest divided equally

gen covwt_50 =.

replace covwt_50= (0.5/(records-1)) if cov_type==0 // half of the weight in contributing causes equally

replace covwt_50= 0.5 if cov_type==1 // half of the weight to covid as ucod

replace covwt_50= 1 if covid==1 & records==1 // all the weight to the ucod if only 1 record

replace covwt_50= 0 if cov_type==.

ta covwt_50 ,m

// all weights divided equally

gen covwt_eq =0

replace covwt_eq= 1/records if anycov==1

ta covwt_eq ,m

**# CHECK WEIGHTS – the sum of weights of each scheme should be 1

//sum up all double weights --> should be 1 for each person

gen checkdouble= L201wt_double + L202wt_double + L203wt_double + L204wt_double + L205wt_double + L206wt_double + L207wt_double + L208wt_double + L209wt_double + ….

//sum up all 50%UC weights --> should be 1 for each person

gen check50= L201wt_50 + L202wt_50 + L203wt_50 + L204wt_50 + L205wt_50 + L206wt_50 + L207wt_50 + L208wt_50 + L209wt_50+ …

//sum up all equal weights --> should be 1 for each person

gen checkeq= L201wt_eq + L202wt_eq + L203wt_eq + L204wt_eq + L205wt_eq + L206wt_eq + L207wt_eq + L208wt_eq + L209wt_eq+ …

tab1 check*,m

1. **Age standardized rates stratified by sex, example for Cancer only**

********************************************************

**# Cancer -UC

********************************************************

// load data

use 20250426colsel.dta, clear

//direct standardization

dstdize L200 pop agegr, by( year sex) using popstd_who.dta print

return list

// Extract results

matrix C = r(crude)', r(adj)', r(lb)' , r(ub)', r(Nobs)', r(se)'

svmat C, names(col)

gen rowname = " "

forval i = 1/8{

replace rowname = r(c`i') in `i'

}

keep Crude Adjusted Left Right Nobs Se rowname

gen year="."

replace year= substr(rowname, 3, 4)

gen sex="."

replace sex= substr(rowname,12, 1)

drop if Se==.

destring year sex, replace

//Amplify

gen crude=Crude*100000

gen adj=Adjusted*100000

gen low=Left*100000

gen upp=Right*100000

label define sex 1 "Men" 2 "Women", modify

label values sex sex

sort year upp low adj

// save for graphs

gen method=1 // identify as UCOD

save CAucstd_sex.dta, replace

********************************************************

* Cancer -MC

********************************************************

// Load data

use 20250426colsel.dta, clear

// round L200wt_503 to closest integer

gen count= round(L200wt_503)

br

// standardize

dstdize count pop agegr, by(year sex) using popstd_who1.dta print

return list

//extract results

matrix C = r(crude)', r(adj)', r(lb)' , r(ub)', r(Nobs)', r(se)'

svmat C, names(col)

gen rowname = " "

forval i = 1/8{

replace rowname = r(c`i') in `i'

}

keep Crude Adjusted Left Right Nobs Se rowname

gen year="."

replace year= substr(rowname, 3, 4)

gen sex="."

replace sex= substr(rowname,12, 1)

drop if Se==.

destring year sex, replace

//amplify

gen crude=Crude*100000

gen adj=Adjusted*100000

gen low=Left*100000

gen upp=Right*100000

label define sex 1 "Men" 2 "Women", modify

label values sex sex

sort year upp low adj

//save for graphs

gen method=2 // to identify as MCOD

save CAmcstd_sex.dta, replace

// visual

use CAmcstd_sex.dta, clear

append using CAucstd_sex.dta

label define sex 1 "Men" 2 "Women", modify

tw ///

(rarea upp low year if sex==1 & method==1, connect(ascending) color(stc1) ylabel(0(50)150, angle(vertical)) ytitle("ASMR (x100,000 pop)") xtitle(" ")) ///

(scatter adj year if sex==1 & method==1, connect(ascending) color(stc1) ) ///

(rarea upp low year if sex==1 & method==2, connect(ascending) color(stc1%60) lpattern(dash)) ///

(scatter adj year if sex==1 & method==2, connect(ascending) color(stc1%60) lpattern(dash) msymbol(X) msize(large)) ///

(rarea upp low year if sex==2 & method==1, connect(ascending) color(stc2)) ///

(scatter adj year if sex==2 & method==1, connect(ascending) color(stc2) ) ///

(rarea upp low year if sex==2 & method==2, connect(ascending) lpattern(dash) color(stc2%80)) ///

(scatter adj year if sex==2 & method==2, connect(ascending) lpattern(dash) msymbol(X) msize(large) color(stc2%80) legend(pos(5) row(1) ring(0) symxsize(6) size(small) order( 2 "Men-UC" 4 "Men-MC" 6 "Women-UC" 8 "Women-MC")) subtitle("A. Colombia", pos(11))), name(CAstd_sexucmc, replace)

graph export "CAstd_sexucmc.png", name(CAstd_sexucmc) width(2000) replace

1. Iris: Iris is a software that automates the coding of causes of death. It uses a dictionary to convert written causes into ICD-10 codes and applies the classification rules to automatically identify the underlying cause of death (UCOD). The dictionary is available in multiple languages and can be adapted as needed. The software is maintained by the Iris Institute, an international collaboration hosted by the Federal Institute for Drugs and Medical Devices (BfArM) in Germany. [↑](#footnote-ref-1)
2. Breger TL, Edwards JK, Cole SR, Saag M, Rebeiro PF, Moore RD, et al. Estimating a set of mortality risk functions with multiple contributing causes of death. Epidemiology. 2020;31:704–12. [↑](#footnote-ref-2)
3. Moreno-Betancur M, Sadaoui H, Piffaretti C, Rey G. Survival analysis with multiple causes of death. Extending the Competing Risks Model. Epidemiology. 2017;28:12–9. [↑](#footnote-ref-3)
